# Supplementary material for: Structural Features and Physiological Associations of Human 14-3-3ζ Pseudogenes
Source: Genes (Basel). 2024 Mar 24;15(4):399. doi: 10.3390/genes15040399 (PMC11049341; doi:10.3390/genes15040399)
Supplement: Supplementary file 1 [file genes-15-00399-s001.zip › genes-2925146-supplementary.pdf]

Supplementary Data

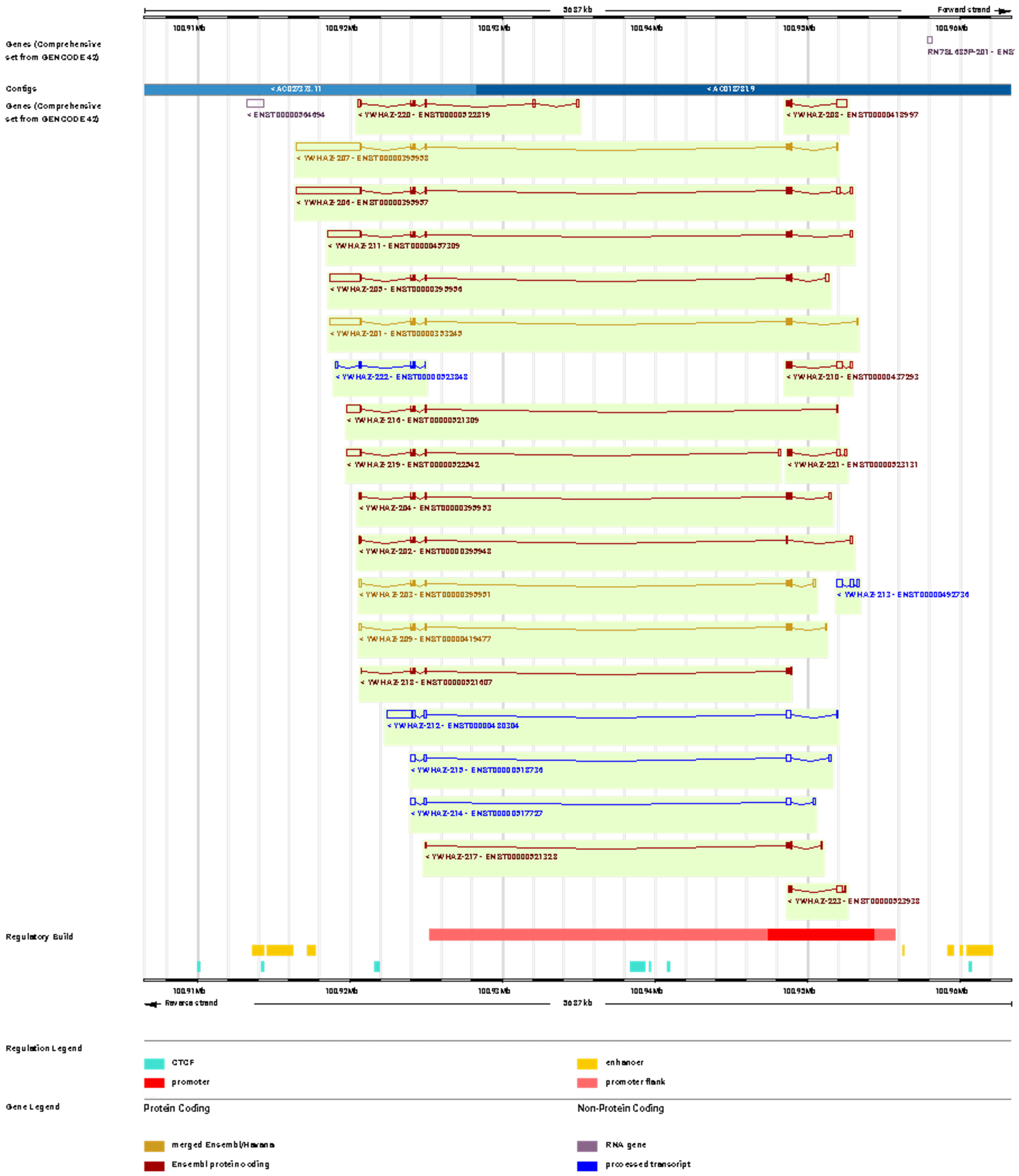

Fig S1: Human *Ywhaz* transcripts from the ensembl portal are shown.

Fig S2: cDNA Alignment [obtained using MView, Reference #29]

```
Reference sequence (1): YWHAZ
Identities normalised by aligned length.
Colored by: identity
```

Key:

u=alignment in small number of sequences
s=alignment in moderate number of sequences

cov = percent coverage of YWHAZ sequence
pid = percent identity in covered regions

```
                     cov    pid    1 [        .         .         .         .         :         .         .         . 80
 1 YWHAZ          100.0% 100.0%      GACAGAGTCTCAGCCTCGCCGCTGCTGCCGCCGCCGCCGCCCAGAGACTGCTGAGCCCGTCCGTCCGCCGCCACCACCCA
 2 YWHAZP1         14.3%  80.6%      --------------------------------------------------------------------------------
 3 YWHAZP2         14.5%  97.1%      --------------------------------------------------------------------------------
 4 YWHAZP3         14.7%  97.6%      --------------------------------------------------------------------------------
 5 YWHAZP4         14.1%  90.5%      --------------------------------------------------------------------------------
 6 YWHAZP5         14.6%  96.0%      --------------------------------------------------------------------------------
 7 YWHAZP6         14.7%  95.1%      --------------------------------------------------------------------------------
 8 YWHAZP7         12.5%  88.4%      --------------------------------------------------------------------------------
 9 YWHAZP8         14.5%  85.2%      --------------------------------------------------------------------------------
10 YWHAZP10        14.7%  98.0%      --------------------------------------------------------------------------------
   consensus/100%                    ................................................................................
   consensus/90%                     ................................................................................
   consensus/80%                     ................................................................................
   consensus/70%                     ................................................................................

                     cov    pid   81          .         1         .         .         .         .         :         . 160
 1 YWHAZ          100.0% 100.0%      CTCCGGACACAGAACATCCAGTCATGGATAAAAATGAGCTGGTTCAGAAGGCCAAACTGGCCGAGCAGGCTGAGCGATAT
 2 YWHAZP1         14.3%  80.6%      -----------------------ATGGGTAAAAATATCCCGGTGCAGAAGGCCAAATGG---------GCTGAGCAATAT
 3 YWHAZP2         14.5%  97.1%      --------------------------GATAAAAATGAGCTGGTTCAGAAGGCCAAACTGGCCGAGCAGGCTGAGCAATAT
 4 YWHAZP3         14.7%  97.6%      -----------------------ATGGATAAAAATGAGCTGGTTCAGAAGGCCAAACTGGCCGAGCAGGCTGAGCGATAT
 5 YWHAZP4         14.1%  90.5%      -----------------------ATGGATAAAAATGAGCTGGTTCAGAAGGCCGAACTGGCCCAGTAGGCTGAGAGATAT
 6 YWHAZP5         14.6%  96.0%      -----------------------ATGGAAAAAAATGAGCTGGTTCAGAAGGCCAAACTGGCTGAGCAGGCTGAGCAATAT
 7 YWHAZP6         14.7%  95.1%      -----------------------ATGGATAAAAATGAGCTGGTTCAGAAGGCCAAACTGGCCGAGCAGGCTGAGTGGTAT
 8 YWHAZP7         12.5%  88.4%      ------------------------------------------------------------------------------AT
 9 YWHAZP8         14.5%  85.2%      -----------------------ATAGATAAATATGAGTTGGTTCAGAAGGCCAAACTAATGGAGCAAGCTGAGAAGTAT
10 YWHAZP10        14.7%  98.0%      -----------------------ATGGATAAAAATGAGCTGGTTCAGAAGGCCAAACTGGCCGAGCAGGCTGAGCGATAT
   consensus/100%                    ..............................................................................AT
   consensus/90%                     ..........................GusAAAsATussssGGTsCAGAAGGCCuAAssu.........GCTGAGsuuTAT
   consensus/80%                     .......................ATuGATAAAAATGAGCTGGTTCAGAAGGCCAAACTGusssAGsAuGCTGAGsuuTAT
   consensus/70%                     .......................ATGGATAAAAATGAGCTGGTTCAGAAGGCCAAACTGGCsGAGCAGGCTGAGsuATAT

                     cov    pid  161          .         .         .         2         .         .         .         . 240
 1 YWHAZ          100.0% 100.0%      GATGACATGGCAGCCTGCATGAAGTCTG-TAACTGAGCAAGGAGCT-GAATTATCCAATG--AGGAGAGGAATCTTCTCT
 2 YWHAZP1         14.3%  80.6%      GTTGTCACGAAAGACTGCATGATGTCTG-TAACTAAGCAAGGAACTGAATTTTTTTTTATTAGGAGAGGAATCTTCTTT
 3 YWHAZP2         14.5%  97.1%      GATGACATGGCAGCCTGCATGAAGTCTG-TAACTAAGCAAGGAGCT-GAATTATCCAATG--AGGAGAGGAATCTTCTCT
 4 YWHAZP3         14.7%  97.6%      GATGACATGGCAGCCTGCATGAAGTCTG-TAACTGAGCAAGGAGCT-GAATTATCCAATG--AGGAAAGGAATCTCCTCT
 5 YWHAZP4         14.1%  90.5%      GATGACATCGCAGCCTGCATGAAATCTG-TAACTGAGCAAGGAGCTGAAATTATCCAATG--AGGAGAGGAAACTTCTCT
 6 YWHAZP5         14.6%  96.0%      GATAACATGGCAGCCTGCATGAAGTCTG-TAACTGAGCAAGGAGCT-GAATTATCCAATG--AGGAGAGGAATCTTCTCT
 7 YWHAZP6         14.7%  95.1%      GATGATATGGCAGCCTGCATGAAGTCTG-TAACTGAGCAAGGAGCT-TAATTATCCAATG--AGGAGAGGAATCTTCTCT
 8 YWHAZP7         12.5%  88.4%      GATGACATGGCAGCCTGCATGAAGTCTTGTAACTGAGTAAGGAGCT-GAATTGTCCAATG--AGGAGAGGAACCTTCTCT
 9 YWHAZP8         14.5%  85.2%      GATGACATGGCAGCCTGCATGAAATCTA-TCACTGAGCAAGGAGAT-GAATTATCCAAGG--AGGAGAGGAATCTTCTTT
10 YWHAZP10        14.7%  98.0%      GATGACATGGCAGCCTGCATGAAGTCTG-TAACTGAGCAAGGAGCT-GAATTATCCAATG--AGGAGAGGAATCTTCTCT
   consensus/100%                    GsTussAssusAGsCTGCATGAsuTCTs.TsACTuAGsAAGGAusT.sAsTTsTsssssu..AGGAuAGGAAsCTsCTsT
   consensus/90%                     GATGACATGGCAGCCTGCATGAAuTCTu.TAACTuAGCAAGGAGCT.uAATTuTCCAATG..AGGAGAGGAAsCTTCTsT
   consensus/80%                     GATGACATGGCAGCCTGCATGAAGTCTG.TAACTGAGCAAGGAGCT.uAATTATCCAATG..AGGAGAGGAATCTTCTCT
   consensus/70%                     GATGACATGGCAGCCTGCATGAAGTCTG.TAACTGAGCAAGGAGCT.GAATTATCCAATG..AGGAGAGGAATCTTCTCT

                     cov    pid  241          :         .         .         .         .         3         .         . 320
 1 YWHAZ          100.0% 100.0%      CAGTTGCTTATAAAAATGTTGTAGGAGCCCGTAGGTCATCTTGGAGGGTCGTCTCAAGTATTGAACAAAAGACGGAAGGT
 2 YWHAZP1         14.3%  80.6%      TAGTTGCTTATAAAAATGTTACAGGAGCCTGTAGGTCATCTTGGAGCTTCGTCTCAAGTATTGGGCAAATGACAGAAG--
 3 YWHAZP2         14.5%  97.1%      CAGTTGCTTATAAAAATGTTGTAGGAGCCCGTAAGTCATCTTGGAGGGTCGTCTCAAGTATTGAACAAAAAACGGAAGGT
 4 YWHAZP3         14.7%  97.6%      CAGTTGCTTATAAAAATGTTGTAGGAGCCCATAGGTCATCTTGGAGGGTCGTCTCAAGTATTGAACAAAAGACGGAGGGA
 5 YWHAZP4         14.1%  90.5%      CAGTTGCTTCTAAAAATG---TAGGCGCCTATAGGTCATTTTGGAGTGTTGTCTCAAGTATTGAACAAAAGACGGAAGGT
 6 YWHAZP5         14.6%  96.0%      CAGTTGCTTATAAAAATGTTGTAGGAGCCCGTAGGTCATCTTGGAGGGTCATCTCAAGTATTGAACAAAAGACGGAAGGT
 7 YWHAZP6         14.7%  95.1%      CAGTTGCTTATAAAAATGTTGTAGGAGCCCGTAGGTCATCTTGGAGCATCGTCTCAAGTATTGAACAAAAGACAGAAGGT
 8 YWHAZP7         12.5%  88.4%      GAGCTGCTTATAAAAATGTTGTAGAAGCCCGTAGATTATCTTGGAGGATCTTCTCAAGTATTGAACAAAAGACGGAAGGT
 9 YWHAZP8         14.5%  85.2%      CAATTACTTATAAAATTGTTGAAGGAGCCCATAGGTCATCTTGGAGGGTCTTTTAAG-TATTAAACAAATGAGGGAAGGT
10 YWHAZP10        14.7%  98.0%      CAGTTGCTTATAAAAATGTTTTAGGAGCCCGTAGGTCATCTTGGAGGGTCGTCTCAAGTATTGAACAAAAGACGGAAGGT
   consensus/100%                    sAusTuCTTsTAAAAsTG...sAGusGCCsuTAuuTsATsTTGGAGssTssTsTsAu.TATTuuuCAAAsuAsuGAuG..
   consensus/90%                     sAGTTGCTTATAAAAATGTTssAGGAGCCsuTAGGTCATCTTGGAGsuTCsTCTCAAGTATTGAACAAAsGACuGAAGGs
   consensus/80%                     CAGTTGCTTATAAAAATGTTuTAGGAGCCCuTAGGTCATCTTGGAGsuTCuTCTCAAGTATTGAACAAAAGACGGAAGGT
   consensus/70%                     CAGTTGCTTATAAAAATGTTGTAGGAGCCCGTAGGTCATCTTGGAGGGTCGTCTCAAGTATTGAACAAAAGACGGAAGGT

                     cov    pid  321          .         .         :         .         .         .         .         4 400
 1 YWHAZ          100.0% 100.0%      GCTGAGAAAAAACAG--CAGATGGCTCGAGAATACAGAGAGAAAATTGAGACGGAGCTAAGAGATATCTGCAATG-ATGT
 2 YWHAZP1         14.3%  80.6%      -CTGCTGAGAAAAACAGCAGATGGCTTGAGAATACAGAGAGAAAATGCAAGTGAGCTAAGAGCTATCTGTAAATGATGT
 3 YWHAZP2         14.5%  97.1%      GCTGAGAAAAAACAG--CAGATGGCTCGAGAACACAGAGAGAAAATTGAGACGGAGCTAAGAGATATCTGTAATG-ATGT
 4 YWHAZP3         14.7%  97.6%      GCTGAGAAAAAACAG--CAGATGGCTCGCGAATACAGAGAGAAAATTGAGACGGAGCTAAGAGATATCTGCAATG-ATGT
 5 YWHAZP4         14.1%  90.5%      GCTGAGAAAAAACAG--CAGATGGCTCGAGAATACAGAGAGAAAATTCAGACAGAGCTAAGAGATATCTGCAATG-ATGT
 6 YWHAZP5         14.6%  96.0%      GTTGAGAAAAAACAG--CAGATGGCTCGAGAATACAGAGAGAAAATTGAGATGGAGCTAAGAGATATCTCCAATG-ATGT
 7 YWHAZP6         14.7%  95.1%      GCTGAGAAAAAAACAG-CAGATGGCTCAAGAATACAGAGAGAAAACTGAGACCTAGCTAAGAGATAGCTGCAATG-ATGT
 8 YWHAZP7         12.5%  88.4%      GCTGAGAATAAAACAGTAGATAGTATGAGAATACAAAGAGAAAATTGAGATGAAGCTAGGAGATATCTTGCAAT-GATT
 9 YWHAZP8         14.5%  85.2%      GCTGAAAAAAAAACAGCAATTAGCTCAAAGTACAGAGATAAAATTGAGACTGAGCTAAGACATATCTGCAATA-AAGC
10 YWHAZP10        14.7%  98.0%      GCTGAGAAAAAACAG--CAGATGGCTCGAGAATACAGAGAGAAAGTTGATACGGAGCTAAGAGATATCTGCAATG-ATGT
   consensus/100%                    .sTGssuAsAAAsss..sAusTuGsssusuAusACAuAGAsAAAusssAsAsssAGCTAuGAssTAsCTsssAss.usss
   consensus/90%                     GCTGAuAAuAAAsAs..CAGATuGCTsuAGAATACAGAGAGAAAATTsAuAssuAGCTAAGAGATATCTssAAss.AsGT
   consensus/80%                     GCTGAGAAAAAAAsAs..CAGATGGCTCGAGAATACAGAGAGAAAATTGAGAssGAGCTAAGAGATATCTGsAATu.ATGT
```

```
    consensus/70%                          GCTGAGAAAAAAsAu..CAGATGGCTCGAGAATACAGAGAGAAAATTGAGACuGAGCTAAGAGATATCTGCAATG.ATGT

                            cov    pid  401         .         .         .         .         :         .         .         . 480
  1 YWHAZ                100.0% 100.0%      ACTGTCTCTTTTGGAAAAGTTCTTGATCCCCAATGCTTCACAAGCAGAGAGCAAAGTCTTCTATTTGAAAATGAAAGGAG
  2 YWHAZP1               14.3%  80.6%      ACTGTCCCTTTTGGAAGAGTTCTTGATCCCCAAAGCTTCACAAGCACAGAGCAAAATCTATTAAAAGCAAAAGGAG----
  3 YWHAZP2               14.5%  97.1%      ATTGTCTCTTTTGGAAAAGTTCTTGATCCCCAATGCTTCACAAGCAGAGAGCAAAGTCTTCTATTTGAAAATGAAAGGAG
  4 YWHAZP3               14.7%  97.6%      ACTGTCTCTTTTGGAAAAGTTCTTGATCCCCAGTGCTTCACAAGCAGAGAGCAAAGTCTTCTATTTGAAAATGGAAGGAG
  5 YWHAZP4               14.1%  90.5%      ACTGTCTCTTTTGGAAAAGTTCTTGATCCCCAGTGCTTCACAAGCAGAGAGCAAAGTCTTCTATTTGAAAATGAAAGGAG
  6 YWHAZP5               14.6%  96.0%      ACTATCTCTTTTGGAAAAGTTCTTGATCTCCAATGCTTCACAAGCAGAGAGCAAAGTCTTCTATTTGAAAATGAAAGGAG
  7 YWHAZP6               14.7%  95.1%      ACTGTCCCTTTTGGAAAAGTTCTTGATCCCCAGTGCTTCACAAGCG-AGAGCAAATTCTTCTATTTGAAAATGAAAGGAG
  8 YWHAZP7               12.5%  88.4%      TACTCCTTTTTTGGAAAAGTTCTTGATCCCCAGTGCTTCACAAGCAGAGAGCAAAGTCTTCTATTTGAAAATGAAAGGAG
  9 YWHAZP8               14.5%  85.2%      ACTATCTCTTTTGGAAATGTACTTGATCCCCAATGCTTCACAG----AGAGCAAAGTCTTCTATTTGAAAATAAAAGGAC
 10 YWHAZP10              14.7%  98.0%      ACTGTTTCTTTTGGAAAAGTTCTTGATCCCCAGTGCTTCACAAGCAGAGAGCAAAGTCTTCTCTTTGAAAATGAAAGGAG
    consensus/100%                         sssssssTTTTGGAAusGTsCTTGATCsCCAusGCTTCACAu....AGAGCAAAsTCTssTssssGsAAAsuuAu....
    consensus/90%                          AsTuTCsCTTTTGGAAAAGTTCTTGATCCCCAuTGCTTCACAAGCu.AGAGCAAAuTCTTCTATTTGAAAATGuAAGGAs
    consensus/80%                          ACTuTCTCTTTTGGAAAAGTTCTTGATCCCCAuTGCTTCACAAGCAsAGAGCAAAGTCTTCTATTTGAAAATGAAAGGAG
    consensus/70%                          ACTGTCTCTTTTGGAAAAGTTCTTGATCCCCAuTGCTTCACAAGCAGAGAGCAAAGTCTTCTATTTGAAAATGAAAGGAG

                            cov    pid  481         .         5         .         .         .         .         :         . 560
  1 YWHAZ                100.0% 100.0%      ATTACTACCGTTACTTGGCTGAGGTTGCCGCTGGTGATGACAAGAAAGGGATTGTCGATCAGTCACAACAAGCATACCAA
  2 YWHAZP1               14.3%  80.6%      TCTACTTTTGTTATTTGGCTCAGGTTGCCGCTAGTGATGATAAGAAAGGGATTGTGGATCAGTCACAACAAGCACATCAA
  3 YWHAZP2               14.5%  97.1%      ATTACTACCGTTACTTGACTGAGGTTACTGCTGGTGATGACAAGATAGGGATTGTGGATCAGTCACAACAAGCATACCAA
  4 YWHAZP3               14.7%  97.6%      ATTACTACCGTTACTTGGCTGAGGTTGCCGCTGGGGATGACAAGAAAGGGATTGTGGATCAGTCACAACAAGCGTACCAA
  5 YWHAZP4               14.1%  90.5%      ATTACTACCGTTACTTGGCTGAGGTCGTTGCTGGTGATGACAAGAAAGGGATTGTGGATCAGTCACAACAAGCATACCAA
  6 YWHAZP5               14.6%  96.0%      ATTACTACCGTTACTTGGCTGAGGTTGCCGCTGGTGATGGCAAGAAAGGGATTGTGGGTCAGTCACAACAAGCATACCAA
  7 YWHAZP6               14.7%  95.1%      ATCACTACCATTACTTGGCTGAGGTTGCCGCTGGTGATGATAAGAAAGGGACTGTGGATCAGTCACAACAAGCACACCAA
  8 YWHAZP7               12.5%  88.4%      ACTACTACGGTTACTTGTCTGAGGTTGCTGCTGGTGATCACAAGAAGGGGATTGTGGATCAGTCACAACAAGCATACCAA
  9 YWHAZP8               14.5%  85.2%      ACTACTATTTTTTTGTAGCTGAGGTTTCTGCTGGTGATGACAAGAAAGAGATTGTGGATCAGTCACAACAAGCATACAGA
 10 YWHAZP10              14.7%  98.0%      ATTACTACCATTACTTGGCAGAGGTTGCCACTGGTGATGACAAGAAAGGGATTGTGGATCAGTCACAACAAGCATACCAA
    consensus/100%                         sssACTssssTTsssTusCssAGGTssssuCTuGsGATsusAAGAsuGuGAsTGTsGuTCAGTCACAACAAGCusAssuA
    consensus/90%                          AsTACTAssuTTAsTTGuCTGAGGTTuCsGCTGGTGATGAsAAGAAAGGGATTGTGGATCAGTCACAACAAGCAsACCAA
    consensus/80%                          AsTACTACsuTTACTTGGCTGAGGTTGCsGCTGGTGATGACAAGAAAGGGATTGTGGATCAGTCACAACAAGCATACCAA
    consensus/70%                          ATTACTACCGTTACTTGGCTGAGGTTGCsGCTGGTGATGACAAGAAAGGGATTGTGGATCAGTCACAACAAGCATACCAA

                            cov    pid  561         .         .         .         6         .         .         .         . 640
  1 YWHAZ                100.0% 100.0%      GAAGCTTTTGAAATCAGCAAAAAGGAAATGCAACCAACACATCCTATCAGACTGGGTCTGGCCCTTAACTTCTCTGTGTT
  2 YWHAZP1               14.3%  80.6%      GAAGCTTTTGAAATCAGAAAAAGG-AATTGCAATCAACACATCCTATCAGATTGGGTCTGGGCTATAAGCTCTCTGTGTT
  3 YWHAZP2               14.5%  97.1%      GAAGCTTTTGAAATCAGCAAAAAAGAAATGCAACCAACACATCCTGTCAGATTGGGTCTGGCCCTTAACTTCTGTG--TT
  4 YWHAZP3               14.7%  97.6%      GAAGCTTTTGAAATCAGCAAAAAGGAAATGCAACCAACACATCCTATCAGACTGGGTCTGGCCCTTAACTTCTCTGTGTT
  5 YWHAZP4               14.1%  90.5%      GAAGCTTTTGAAATCAGCAAAAAGGAAATGCAACCAACACATGCTATCAGACTGG-------------------------
  6 YWHAZP5               14.6%  96.0%      GAAGCTTTTGAAATCAGCAAAAAGGAAATGCAACCAACACATACTGTCAGACTGGGTCTGGCCCTTAACTTCTCTTG-TT
  7 YWHAZP6               14.7%  95.1%      GAAGCTTTTGAAATCAGCAAAAAGGAAATGCAACCAACATATCCTATCAGACTGGGTCTGGCCCTTAACTTCTCCGTGTT
  8 YWHAZP7               12.5%  88.4%      GAAGCTTTTGGAATCAGCAAAAAGGAAATGCAACGAGCACATCCTATCAGATTGGGGCTGGCCCTTAACTACTCTGTGTT
  9 YWHAZP8               14.5%  85.2%      GAAGCTTTTGAAATTAGCAAAAAGGAAATGCAACCAACATATCCTACCAGATTGGGTCTGGCCTTTAATTTTTCTGTATT
 10 YWHAZP10              14.7%  98.0%      GAAGCTTTTGAAATCAGCAAAAAGGAAATGCAACCAACACATCCTATCAGACCAGGTCTGGCCCTTAACTTCTCTGTGTT
    consensus/100%                         GAAGCTTTTGuAATsAGsAAAAuu.AAsTGCAAssAuCAsATsCTusCAGAssuG.........................
    consensus/90%                          GAAGCTTTTGAAATCAGCAAAAAGGAAATGCAACCAACAsATsCTuTCAGAsTGGGsCTGGsCssTAAssssTsss..TT
    consensus/80%                          GAAGCTTTTGAAATCAGCAAAAAGGAAATGCAACCAACACATCCTATCAGAsTGGGTCTGGCCsTTAAsTTCTCTGs.TT
    consensus/70%                          GAAGCTTTTGAAATCAGCAAAAAGGAAATGCAACCAACACATCCTATCAGAsTGGGTCTGGCCCTTAACTTCTCTGTuTT

                            cov    pid  641         :         .         .         .         .         7         .         . 720
  1 YWHAZ                100.0% 100.0%      CTATTATGAGATTCTGAACTCCCCAG-AGAAAGCCTGCTCTCTTGCAAAGACAGCTTTTGATGAAGCCATTGCTGAACTT
  2 YWHAZP1               14.3%  80.6%      GTATTGTGAGAATCTGAACTTGCTGG-AGAAAGCCTGCTGTCTTGCAAAGACAGCTTTTGATGAAGCCATTGTTGGACTC
  3 YWHAZP2               14.5%  97.1%      CTATTATGAGATTCTGAACTCCCCAG-AGAAAGCCTGCTGTCTTGCAAAGACCGCTTTTGATGAAGCCATTGCTGAACTT
  4 YWHAZP3               14.7%  97.6%      CTATTATGAGATTCTGAACTCCCCAG-AGAAAGCCTGCTCTCTTGCAAAGACAGCTTTTGATGAAGCCATTGCTGAACTT
  5 YWHAZP4               14.1%  90.5%      ---ATTTACTCCTCTGAACTCCCCAG-GGAAAGCCTGTTCTCTTGCAAAGACAGCTTTTGATGAAGCCATTGCTGAACTT
  6 YWHAZP5               14.6%  96.0%      CTATTATGAGATTCTGAACTCCCCAG-ATAAAGTCTGCTCTCTTGCAAAGATGGCTTTTGATGAAGGCATTGCTGAACTT
  7 YWHAZP6               14.7%  95.1%      CTATTATGACGTTCTGAATTCCCCAG-AGAAAGCCTGCTCTCTTGCAAAGACAGCTTTTGATGAAGCCATTGCTGAACTT
  8 YWHAZP7               12.5%  88.4%      TTATTATGAGATTCTGAACTCCCCCAGAGAAAGCCTGCTGTCTTGCAAAGACAGCTGCCAATGAAGCTATTGCTGAACTT
  9 YWHAZP8               14.5%  85.2%      CCATTATGAGATTCTAAACTCCCCAG-ATAAAGCCTGCTCTCTTACAAGGACAGCTTTTGATAAAGCCATTGCTGAATTT
 10 YWHAZP10              14.7%  98.0%      CTATTATGAGATTCTGAACTCCCCAG-AGAAAGCCTGCTCTCTTGCAAAGACAGCTTTTGATGAAGCCATTGCTGAACTT
    consensus/100%                         ...sTsTussssTCTuAAsTssCssu.usAAAGsCTGsTsTCTTuCAAuGAssGCTsssuATuAAGssATTGsTGuAsTs
    consensus/90%                          ssATTuTGAsusTCTGAACTCCCCuG.AsAAAGCCTGCTsTCTTGCAAAGACuGCTTTTGATGAAGCCATTGCTGAACTT
    consensus/80%                          sTATTATGAGATTCTGAACTCCCCAG.AGAAAGCCTGCTsTCTTGCAAAGACAGCTTTTGATGAAGCCATTGCTGAACTT
    consensus/70%                          CTATTATGAGATTCTGAACTCCCCAG.AGAAAGCCTGCTCTCTTGCAAAGACAGCTTTTGATGAAGCCATTGCTGAACTT

                            cov    pid  721         .         .         :         .         .         .         .         8 800
  1 YWHAZ                100.0% 100.0%      GATACATTAAGTGAAGAGTCATACAAAGACAGCACGCTAATAATGCAATTACTGAGAGACAACTTGACATTGTGGACATC
  2 YWHAZP1               14.3%  80.6%      AATACATTAAGAG-----TCATATAAAGATAGCATGCTAATAATGCAACTACTGAGAAACTACTTAACATTTTACACATC
  3 YWHAZP2               14.5%  97.1%      GATACATTAAGTGAAGAGTCATACAAAGACAGCATGCTAATAATGCAATTACTGAGAGACAACTTGACATTGTGGACATC
  4 YWHAZP3               14.7%  97.6%      GATACATTAAGTGAAGAGTCATACAAAGACAGCACGCTAATAATGCAATTACTGAGGGACAACCTGACACTGTGGACATC
  5 YWHAZP4               14.1%  90.5%      GATATATTAAGTGAAGAGTCATACAAAGACAACACGCTAATAATGCAGTTACTGACAGACAACTTGACATTGTGGACATC
  6 YWHAZP5               14.6%  96.0%      GATACATTAAATGAAGAGTCATACAAAGACAGCACGCTAATAATGCAATTACCGAGAGACAACTTGACATTGTGGACACT
  7 YWHAZP6               14.7%  95.1%      GATACATTAAGTGAAGAGTCATCCAAAGACAGCACACTAATAATGCAATTACTGAGAGACAACTTGACATTGTGGACATC
  8 YWHAZP7               12.5%  88.4%      GATACATTAAGT-------CATACAAAGATAGCACGCTAATAATGCAATTACTGATAGACAACTCGACATTGTGGACATC
  9 YWHAZP8               14.5%  85.2%      GACACATTTAGCACAGAGCCATACAAAGACAGCACAGTAGTTTTGAAACTACTGAGAAATGGCTTGACATTGCAGACATC
 10 YWHAZP10              14.7%  98.0%      GATACATTAATTGAAGAGTCATACAAAGACAGCATGCTAATAATGCAATTACTGAGAGACAACTTGACATTGTGGACATC
    consensus/100%                         uAsAsATTsAss.......CATssAAAGAsAuCAsusTAuTssTGsAusTACsGAsuuAssuCssuACAsTssusACAss
    consensus/90%                          GATACATTAAusu.....sCATACAAAGAsAGCAsuCTAATAATGCAAsTACTGAsAuACuACTTGACATTGTuGACATC
    consensus/80%                          GATACATTAAGTGsAGAGTCATACAAAGACAGCAsGCTAATAATGCAATTACTGAGAGACAACTTGACATTGTGGACATC
    consensus/70%                          GATACATTAAGTGAAGAGTCATACAAAGACAGCACGCTAATAATGCAATTACTGAGAGACAACTTGACATTGTGGACATC

                            cov    pid  801         .         .         .         .         :         .         .         . 880
  1 YWHAZ                100.0% 100.0%      GGATACCCAAGGAGACGAAGCTGAAGCAGGAGAAGGAGGGGAAAATTAACCGGCCTTCCAACTTTTGTCTGCCTCATTCT
  2 YWHAZP1               14.3%  80.6%      AGATACCCAAGGAGATGAAGCTGAAGCAGGAGAAGAAGGTGAAAATTAA-------------------------------
```

```
 3 YWHAZP2         14.5%  97.1%      GGATACCCAAGGAGACGAAGCTGAAGCAGGAGAAGGAGGGGAAA------------------------------------
 4 YWHAZP3         14.7%  97.6%      AGATACCCAAGCAGACGAAGGTGAAGCAGGAGAAGGAGTGGAAAATTAA-------------------------------
 5 YWHAZP4         14.1%  90.5%      GGATACCCAAGGAGACAAAGCTGAAGCAGGAGAAGGAGGTGAAAATTAA-------------------------------
 6 YWHAZP5         14.6%  96.0%      GGATACCCAAGGAGACAAAGCTGAAGCAGGAGAAAGAGGGGA--------------------------------------
 7 YWHAZP6         14.7%  95.1%      GGATACCCGAGGAGACAAAGCTGAAGCAGGAGAAGGAGGGGAAAATTAA-------------------------------
 8 YWHAZP7         12.5%  88.4%      --------------------------------------------------------------------------------
 9 YWHAZP8         14.5%  85.2%      AGATACCCAAGGAGATGAAGCTGAAGCAAGAGAAGGAGGAGAAAA-----------------------------------
10 YWHAZP10        14.7%  98.0%      AGATACCCAAGGAGACGAAGCTGAAGCAGGAGAAGGAGGGGAAAAT----------------------------------
   consensus/100%                    ................................................................................
   consensus/90%                     uGATACCCuAGsAGAsuAAGsTGAAGCAuGAGAAuuAGssGA......................................
   consensus/80%                     uGATACCCAAGGAGAsuAAGCTGAAGCAGGAGAAGGAGGsGAAA....................................
   consensus/70%                     uGATACCCAAGGAGACuAAGCTGAAGCAGGAGAAGGAGGuGAAAA...................................

                     cov    pid  881          .         9         .         .         .         .         :         . 960
 1 YWHAZ          100.0% 100.0%      AAAATTTACACAGTAGACCATTTGTCATCCATGCTGTCCCACAAATAGTTTTTTGTTTACGATTTATGACAGGTTTATGT
 2 YWHAZP1         14.3%  80.6%      --------------------------------------------------------------------------------
 3 YWHAZP2         14.5%  97.1%      --------------------------------------------------------------------------------
 4 YWHAZP3         14.7%  97.6%      --------------------------------------------------------------------------------
 5 YWHAZP4         14.1%  90.5%      --------------------------------------------------------------------------------
 6 YWHAZP5         14.6%  96.0%      --------------------------------------------------------------------------------
 7 YWHAZP6         14.7%  95.1%      --------------------------------------------------------------------------------
 8 YWHAZP7         12.5%  88.4%      --------------------------------------------------------------------------------
 9 YWHAZP8         14.5%  85.2%      --------------------------------------------------------------------------------
10 YWHAZP10        14.7%  98.0%      --------------------------------------------------------------------------------
   consensus/100%                    ................................................................................
   consensus/90%                     ................................................................................
   consensus/80%                     ................................................................................
   consensus/70%                     ................................................................................

                     cov    pid  961          .         .         .         0         .         .         .         . 1040
 1 YWHAZ          100.0% 100.0%      TACTTCTATTTGAATTTCTATATTTCCCATGTGGTTTTTATGTTTAATATTAGGGGAGTAGAGCCAGTTAACATTTAGGG
 2 YWHAZP1         14.3%  80.6%      --------------------------------------------------------------------------------
 3 YWHAZP2         14.5%  97.1%      --------------------------------------------------------------------------------
 4 YWHAZP3         14.7%  97.6%      --------------------------------------------------------------------------------
 5 YWHAZP4         14.1%  90.5%      --------------------------------------------------------------------------------
 6 YWHAZP5         14.6%  96.0%      --------------------------------------------------------------------------------
 7 YWHAZP6         14.7%  95.1%      --------------------------------------------------------------------------------
 8 YWHAZP7         12.5%  88.4%      --------------------------------------------------------------------------------
 9 YWHAZP8         14.5%  85.2%      --------------------------------------------------------------------------------
10 YWHAZP10        14.7%  98.0%      --------------------------------------------------------------------------------
   consensus/100%                    ................................................................................
   consensus/90%                     ................................................................................
   consensus/80%                     ................................................................................
   consensus/70%                     ................................................................................

                     cov    pid 1041          :         .         .         .         .         1         .         . 1120
 1 YWHAZ          100.0% 100.0%      AGTTATCTGTTTTCATCTTGAGGTGGCCAATATGGGGATGTGGAATTTTTATACAAGTTATAAGTGTTTGGCATAGTACT
 2 YWHAZP1         14.3%  80.6%      --------------------------------------------------------------------------------
 3 YWHAZP2         14.5%  97.1%      --------------------------------------------------------------------------------
 4 YWHAZP3         14.7%  97.6%      --------------------------------------------------------------------------------
 5 YWHAZP4         14.1%  90.5%      --------------------------------------------------------------------------------
 6 YWHAZP5         14.6%  96.0%      --------------------------------------------------------------------------------
 7 YWHAZP6         14.7%  95.1%      --------------------------------------------------------------------------------
 8 YWHAZP7         12.5%  88.4%      --------------------------------------------------------------------------------
 9 YWHAZP8         14.5%  85.2%      --------------------------------------------------------------------------------
10 YWHAZP10        14.7%  98.0%      --------------------------------------------------------------------------------
   consensus/100%                    ................................................................................
   consensus/90%                     ................................................................................
   consensus/80%                     ................................................................................
   consensus/70%                     ................................................................................

                     cov    pid 1121          .         .         :         .         .         .         .         2 1200
 1 YWHAZ          100.0% 100.0%      TTTGGTACATTGTGGCTTCAAAAGGGCCAGTGTAAAACTGCTTCCATGTCTAAGCAAAGAAAACTGCCTACATACTGGTT
 2 YWHAZP1         14.3%  80.6%      --------------------------------------------------------------------------------
 3 YWHAZP2         14.5%  97.1%      --------------------------------------------------------------------------------
 4 YWHAZP3         14.7%  97.6%      --------------------------------------------------------------------------------
 5 YWHAZP4         14.1%  90.5%      --------------------------------------------------------------------------------
 6 YWHAZP5         14.6%  96.0%      --------------------------------------------------------------------------------
 7 YWHAZP6         14.7%  95.1%      --------------------------------------------------------------------------------
 8 YWHAZP7         12.5%  88.4%      --------------------------------------------------------------------------------
 9 YWHAZP8         14.5%  85.2%      --------------------------------------------------------------------------------
10 YWHAZP10        14.7%  98.0%      --------------------------------------------------------------------------------
   consensus/100%                    ................................................................................
   consensus/90%                     ................................................................................
   consensus/80%                     ................................................................................
   consensus/70%                     ................................................................................

                     cov    pid 1201          .         .         .         .         :         .         .         . 1280
 1 YWHAZ          100.0% 100.0%      TGTCCTGGCGGGGAATAAAAGGGATCATTGGTTCCAGTCACAGGTGTAGTAATTGTGGGTACTTTAAGGTTTGGAGCACT
 2 YWHAZP1         14.3%  80.6%      --------------------------------------------------------------------------------
 3 YWHAZP2         14.5%  97.1%      --------------------------------------------------------------------------------
 4 YWHAZP3         14.7%  97.6%      --------------------------------------------------------------------------------
 5 YWHAZP4         14.1%  90.5%      --------------------------------------------------------------------------------
 6 YWHAZP5         14.6%  96.0%      --------------------------------------------------------------------------------
 7 YWHAZP6         14.7%  95.1%      --------------------------------------------------------------------------------
```

```
  8 YWHAZP7         12.5%  88.4%      --------------------------------------------------------------------------------
  9 YWHAZP8         14.5%  85.2%      --------------------------------------------------------------------------------
 10 YWHAZP10        14.7%  98.0%      --------------------------------------------------------------------------------
    consensus/100%                    ................................................................................
    consensus/90%                     ................................................................................
    consensus/80%                     ................................................................................
    consensus/70%                     ................................................................................

                      cov     pid 1281         .         3         .         .         .         .         :         . 1360
  1 YWHAZ          100.0% 100.0%      TACAAGGCTGTGGTAGAATCATACCCCATGGATACCACATATTAAACCATGTATATCTGTGGAATACTCAATGTGTACAC
  2 YWHAZP1         14.3%  80.6%      --------------------------------------------------------------------------------
  3 YWHAZP2         14.5%  97.1%      --------------------------------------------------------------------------------
  4 YWHAZP3         14.7%  97.6%      --------------------------------------------------------------------------------
  5 YWHAZP4         14.1%  90.5%      --------------------------------------------------------------------------------
  6 YWHAZP5         14.6%  96.0%      --------------------------------------------------------------------------------
  7 YWHAZP6         14.7%  95.1%      --------------------------------------------------------------------------------
  8 YWHAZP7         12.5%  88.4%      --------------------------------------------------------------------------------
  9 YWHAZP8         14.5%  85.2%      --------------------------------------------------------------------------------
 10 YWHAZP10        14.7%  98.0%      --------------------------------------------------------------------------------
    consensus/100%                    ................................................................................
    consensus/90%                     ................................................................................
    consensus/80%                     ................................................................................
    consensus/70%                     ................................................................................

                      cov     pid 1361         .         .         .         4         .         .         .         . 1440
  1 YWHAZ          100.0% 100.0%      CTTTGACTACAGCTGCAGAAGTGTTCCTTTAGACAAAGTTGTGACCCATTTTACTCTGGATAAGGGCAGAAACGGTTCAC
  2 YWHAZP1         14.3%  80.6%      --------------------------------------------------------------------------------
  3 YWHAZP2         14.5%  97.1%      --------------------------------------------------------------------------------
  4 YWHAZP3         14.7%  97.6%      --------------------------------------------------------------------------------
  5 YWHAZP4         14.1%  90.5%      --------------------------------------------------------------------------------
  6 YWHAZP5         14.6%  96.0%      --------------------------------------------------------------------------------
  7 YWHAZP6         14.7%  95.1%      --------------------------------------------------------------------------------
  8 YWHAZP7         12.5%  88.4%      --------------------------------------------------------------------------------
  9 YWHAZP8         14.5%  85.2%      --------------------------------------------------------------------------------
 10 YWHAZP10        14.7%  98.0%      --------------------------------------------------------------------------------
    consensus/100%                    ................................................................................
    consensus/90%                     ................................................................................
    consensus/80%                     ................................................................................
    consensus/70%                     ................................................................................

                      cov     pid 1441         :         .         .         .         .         5         .         . 1520
  1 YWHAZ          100.0% 100.0%      ATTCCATTATTTGTAAAGTTACCTGCTGTTAGCTTTCATTATTTTTGCTACACTCATTTTATTTGTATTTAAATGTTTTA
  2 YWHAZP1         14.3%  80.6%      --------------------------------------------------------------------------------
  3 YWHAZP2         14.5%  97.1%      --------------------------------------------------------------------------------
  4 YWHAZP3         14.7%  97.6%      --------------------------------------------------------------------------------
  5 YWHAZP4         14.1%  90.5%      --------------------------------------------------------------------------------
  6 YWHAZP5         14.6%  96.0%      --------------------------------------------------------------------------------
  7 YWHAZP6         14.7%  95.1%      --------------------------------------------------------------------------------
  8 YWHAZP7         12.5%  88.4%      --------------------------------------------------------------------------------
  9 YWHAZP8         14.5%  85.2%      --------------------------------------------------------------------------------
 10 YWHAZP10        14.7%  98.0%      --------------------------------------------------------------------------------
    consensus/100%                    ................................................................................
    consensus/90%                     ................................................................................
    consensus/80%                     ................................................................................
    consensus/70%                     ................................................................................

                      cov     pid 1521         .         .         :         .         .         .         .         6 1600
  1 YWHAZ          100.0% 100.0%      GGCAACCTAAGAACAAATGTAAAAGTAAAGATGCAGGAAAAATGAATTGCTTGGTATTCATTACTTCATGTATATCAAGC
  2 YWHAZP1         14.3%  80.6%      --------------------------------------------------------------------------------
  3 YWHAZP2         14.5%  97.1%      --------------------------------------------------------------------------------
  4 YWHAZP3         14.7%  97.6%      --------------------------------------------------------------------------------
  5 YWHAZP4         14.1%  90.5%      --------------------------------------------------------------------------------
  6 YWHAZP5         14.6%  96.0%      --------------------------------------------------------------------------------
  7 YWHAZP6         14.7%  95.1%      --------------------------------------------------------------------------------
  8 YWHAZP7         12.5%  88.4%      --------------------------------------------------------------------------------
  9 YWHAZP8         14.5%  85.2%      --------------------------------------------------------------------------------
 10 YWHAZP10        14.7%  98.0%      --------------------------------------------------------------------------------
    consensus/100%                    ................................................................................
    consensus/90%                     ................................................................................
    consensus/80%                     ................................................................................
    consensus/70%                     ................................................................................

                      cov     pid 1601         .         .         .         .         :         .         .         . 1680
  1 YWHAZ          100.0% 100.0%      ACAGCAGTAAAACAAAAACCCATGTATTTAACTTTTTTTTAGGATTTTTGCTTTTGTGATTTTTTTTTTTTTGATACTTG
  2 YWHAZP1         14.3%  80.6%      --------------------------------------------------------------------------------
  3 YWHAZP2         14.5%  97.1%      --------------------------------------------------------------------------------
  4 YWHAZP3         14.7%  97.6%      --------------------------------------------------------------------------------
  5 YWHAZP4         14.1%  90.5%      --------------------------------------------------------------------------------
  6 YWHAZP5         14.6%  96.0%      --------------------------------------------------------------------------------
  7 YWHAZP6         14.7%  95.1%      --------------------------------------------------------------------------------
  8 YWHAZP7         12.5%  88.4%      --------------------------------------------------------------------------------
  9 YWHAZP8         14.5%  85.2%      --------------------------------------------------------------------------------
 10 YWHAZP10        14.7%  98.0%      --------------------------------------------------------------------------------
    consensus/100%                    ................................................................................
    consensus/90%                     ................................................................................
```

```
    consensus/80%                   ................................................................................
    consensus/70%                   ................................................................................

                    cov     pid 1681         .         7         .         .         .         .         :         . 1760
  1 YWHAZ        100.0%  100.0%     CCTAACATGCATGTGCTGTAAAAATAGTTAACAGGGAAATAACTTGAGATGATGGCTAGCTTTGTTTAATGTCTTATGAA
  2 YWHAZP1       14.3%   80.6%     --------------------------------------------------------------------------------
  3 YWHAZP2       14.5%   97.1%     --------------------------------------------------------------------------------
  4 YWHAZP3       14.7%   97.6%     --------------------------------------------------------------------------------
  5 YWHAZP4       14.1%   90.5%     --------------------------------------------------------------------------------
  6 YWHAZP5       14.6%   96.0%     --------------------------------------------------------------------------------
  7 YWHAZP6       14.7%   95.1%     --------------------------------------------------------------------------------
  8 YWHAZP7       12.5%   88.4%     --------------------------------------------------------------------------------
  9 YWHAZP8       14.5%   85.2%     --------------------------------------------------------------------------------
 10 YWHAZP10      14.7%   98.0%     --------------------------------------------------------------------------------
    consensus/100%                  ................................................................................
    consensus/90%                   ................................................................................
    consensus/80%                   ................................................................................
    consensus/70%                   ................................................................................

                    cov     pid 1761         .         .         .         8         .         .         .         . 1840
  1 YWHAZ        100.0%  100.0%     ATTTTCATGAACAATCCAAGCATAATTGTTAAGAACACGTGTATTAAATTCATGTAAGTGGAATAAAAGTTTTATGAATG
  2 YWHAZP1       14.3%   80.6%     --------------------------------------------------------------------------------
  3 YWHAZP2       14.5%   97.1%     --------------------------------------------------------------------------------
  4 YWHAZP3       14.7%   97.6%     --------------------------------------------------------------------------------
  5 YWHAZP4       14.1%   90.5%     --------------------------------------------------------------------------------
  6 YWHAZP5       14.6%   96.0%     --------------------------------------------------------------------------------
  7 YWHAZP6       14.7%   95.1%     --------------------------------------------------------------------------------
  8 YWHAZP7       12.5%   88.4%     --------------------------------------------------------------------------------
  9 YWHAZP8       14.5%   85.2%     --------------------------------------------------------------------------------
 10 YWHAZP10      14.7%   98.0%     --------------------------------------------------------------------------------
    consensus/100%                  ................................................................................
    consensus/90%                   ................................................................................
    consensus/80%                   ................................................................................
    consensus/70%                   ................................................................................

                    cov     pid 1841         :         .         .         .         .         9         .         . 1920
  1 YWHAZ        100.0%  100.0%     GACTTTTCAACTACTTTCTCTACAGCTTTTCATGTAAATTAGTCTTGGTTCTGAAACTTCTCTAAAGGAAATTGTACATT
  2 YWHAZP1       14.3%   80.6%     --------------------------------------------------------------------------------
  3 YWHAZP2       14.5%   97.1%     --------------------------------------------------------------------------------
  4 YWHAZP3       14.7%   97.6%     --------------------------------------------------------------------------------
  5 YWHAZP4       14.1%   90.5%     --------------------------------------------------------------------------------
  6 YWHAZP5       14.6%   96.0%     --------------------------------------------------------------------------------
  7 YWHAZP6       14.7%   95.1%     --------------------------------------------------------------------------------
  8 YWHAZP7       12.5%   88.4%     --------------------------------------------------------------------------------
  9 YWHAZP8       14.5%   85.2%     --------------------------------------------------------------------------------
 10 YWHAZP10      14.7%   98.0%     --------------------------------------------------------------------------------
    consensus/100%                  ................................................................................
    consensus/90%                   ................................................................................
    consensus/80%                   ................................................................................
    consensus/70%                   ................................................................................

                    cov     pid 1921         .         .         :         .         .         .         .         0 2000
  1 YWHAZ        100.0%  100.0%     TTTTGAAATTTATTCCTTATTCCCTCTTGGCAGCTAATGGGCTCTTACCAAGTTTAAACACAAAATTTATCATAACAAAA
  2 YWHAZP1       14.3%   80.6%     --------------------------------------------------------------------------------
  3 YWHAZP2       14.5%   97.1%     --------------------------------------------------------------------------------
  4 YWHAZP3       14.7%   97.6%     --------------------------------------------------------------------------------
  5 YWHAZP4       14.1%   90.5%     --------------------------------------------------------------------------------
  6 YWHAZP5       14.6%   96.0%     --------------------------------------------------------------------------------
  7 YWHAZP6       14.7%   95.1%     --------------------------------------------------------------------------------
  8 YWHAZP7       12.5%   88.4%     --------------------------------------------------------------------------------
  9 YWHAZP8       14.5%   85.2%     --------------------------------------------------------------------------------
 10 YWHAZP10      14.7%   98.0%     --------------------------------------------------------------------------------
    consensus/100%                  ................................................................................
    consensus/90%                   ................................................................................
    consensus/80%                   ................................................................................
    consensus/70%                   ................................................................................

                    cov     pid 2001         .         .         .         .         :         .         .         . 2080
  1 YWHAZ        100.0%  100.0%     ATACTACTAATATAACTACTGTTTCCATGTCCCATGATCCCCTCTCTTCCTCCCCACCCTGAAAAAAATGAGTTCCTATT
  2 YWHAZP1       14.3%   80.6%     --------------------------------------------------------------------------------
  3 YWHAZP2       14.5%   97.1%     --------------------------------------------------------------------------------
  4 YWHAZP3       14.7%   97.6%     --------------------------------------------------------------------------------
  5 YWHAZP4       14.1%   90.5%     --------------------------------------------------------------------------------
  6 YWHAZP5       14.6%   96.0%     --------------------------------------------------------------------------------
  7 YWHAZP6       14.7%   95.1%     --------------------------------------------------------------------------------
  8 YWHAZP7       12.5%   88.4%     --------------------------------------------------------------------------------
  9 YWHAZP8       14.5%   85.2%     --------------------------------------------------------------------------------
 10 YWHAZP10      14.7%   98.0%     --------------------------------------------------------------------------------
    consensus/100%                  ................................................................................
    consensus/90%                   ................................................................................
    consensus/80%                   ................................................................................
    consensus/70%                   ................................................................................

                    cov     pid 2081         .         1         .         .         .         .         :         . 2160
  1 YWHAZ        100.0%  100.0%     TTTTCTGGGAGAGGGGGGGATTGATTAGAAAAAAATGTAGTGTGTTCCATTTAAAATTTTGGCATATGGCATTTTCTAAC
```

```
  2 YWHAZP1         14.3%  80.6%      --------------------------------------------------------------------------------
  3 YWHAZP2         14.5%  97.1%      --------------------------------------------------------------------------------
  4 YWHAZP3         14.7%  97.6%      --------------------------------------------------------------------------------
  5 YWHAZP4         14.1%  90.5%      --------------------------------------------------------------------------------
  6 YWHAZP5         14.6%  96.0%      --------------------------------------------------------------------------------
  7 YWHAZP6         14.7%  95.1%      --------------------------------------------------------------------------------
  8 YWHAZP7         12.5%  88.4%      --------------------------------------------------------------------------------
  9 YWHAZP8         14.5%  85.2%      --------------------------------------------------------------------------------
 10 YWHAZP10        14.7%  98.0%      --------------------------------------------------------------------------------
    consensus/100%                    ................................................................................
    consensus/90%                     ................................................................................
    consensus/80%                     ................................................................................
    consensus/70%                     ................................................................................

                      cov    pid 2161          .         .         .         2         .         .         .         . 2240
  1 YWHAZ          100.0% 100.0%      TTAGGAAGCCACAATGTTCTTGGCCCATCATGACATTGGGTAGCATTAACTGTAAGTTTTGTGCTTCCAAATCACTTTTT
  2 YWHAZP1         14.3%  80.6%      --------------------------------------------------------------------------------
  3 YWHAZP2         14.5%  97.1%      --------------------------------------------------------------------------------
  4 YWHAZP3         14.7%  97.6%      --------------------------------------------------------------------------------
  5 YWHAZP4         14.1%  90.5%      --------------------------------------------------------------------------------
  6 YWHAZP5         14.6%  96.0%      --------------------------------------------------------------------------------
  7 YWHAZP6         14.7%  95.1%      --------------------------------------------------------------------------------
  8 YWHAZP7         12.5%  88.4%      --------------------------------------------------------------------------------
  9 YWHAZP8         14.5%  85.2%      --------------------------------------------------------------------------------
 10 YWHAZP10        14.7%  98.0%      --------------------------------------------------------------------------------
    consensus/100%                    ................................................................................
    consensus/90%                     ................................................................................
    consensus/80%                     ................................................................................
    consensus/70%                     ................................................................................

                      cov    pid 2241          :         .         .         .         .         3         .         . 2320
  1 YWHAZ          100.0% 100.0%      GGTTTTTAAGAATTTCTTGATACTCTTATAGCCTGCCTTCAATTTTGATCCTTTATTCTTTCTATTTGTCAGGTGCACAA
  2 YWHAZP1         14.3%  80.6%      --------------------------------------------------------------------------------
  3 YWHAZP2         14.5%  97.1%      --------------------------------------------------------------------------------
  4 YWHAZP3         14.7%  97.6%      --------------------------------------------------------------------------------
  5 YWHAZP4         14.1%  90.5%      --------------------------------------------------------------------------------
  6 YWHAZP5         14.6%  96.0%      --------------------------------------------------------------------------------
  7 YWHAZP6         14.7%  95.1%      --------------------------------------------------------------------------------
  8 YWHAZP7         12.5%  88.4%      --------------------------------------------------------------------------------
  9 YWHAZP8         14.5%  85.2%      --------------------------------------------------------------------------------
 10 YWHAZP10        14.7%  98.0%      --------------------------------------------------------------------------------
    consensus/100%                    ................................................................................
    consensus/90%                     ................................................................................
    consensus/80%                     ................................................................................
    consensus/70%                     ................................................................................

                      cov    pid 2321          .         .         :         .         .         .         .         4 2400
  1 YWHAZ          100.0% 100.0%      GATTACCTTCCTGTTTTAGCCTTCTGTCTTGTCACCAACCATTCTTACTTGGTGGCCATGTACTTGGAAAAAGGCCGCAT
  2 YWHAZP1         14.3%  80.6%      --------------------------------------------------------------------------------
  3 YWHAZP2         14.5%  97.1%      --------------------------------------------------------------------------------
  4 YWHAZP3         14.7%  97.6%      --------------------------------------------------------------------------------
  5 YWHAZP4         14.1%  90.5%      --------------------------------------------------------------------------------
  6 YWHAZP5         14.6%  96.0%      --------------------------------------------------------------------------------
  7 YWHAZP6         14.7%  95.1%      --------------------------------------------------------------------------------
  8 YWHAZP7         12.5%  88.4%      --------------------------------------------------------------------------------
  9 YWHAZP8         14.5%  85.2%      --------------------------------------------------------------------------------
 10 YWHAZP10        14.7%  98.0%      --------------------------------------------------------------------------------
    consensus/100%                    ................................................................................
    consensus/90%                     ................................................................................
    consensus/80%                     ................................................................................
    consensus/70%                     ................................................................................

                      cov    pid 2401          .         .         .         .         :         .         .         . 2480
  1 YWHAZ          100.0% 100.0%      GATCTTTCTGGCTCCACTCAGTGTCTAAGGCACCCTGCTTCCTTTGCTTGCATCCCACAGACTATTTCCCTCATCCTATT
  2 YWHAZP1         14.3%  80.6%      --------------------------------------------------------------------------------
  3 YWHAZP2         14.5%  97.1%      --------------------------------------------------------------------------------
  4 YWHAZP3         14.7%  97.6%      --------------------------------------------------------------------------------
  5 YWHAZP4         14.1%  90.5%      --------------------------------------------------------------------------------
  6 YWHAZP5         14.6%  96.0%      --------------------------------------------------------------------------------
  7 YWHAZP6         14.7%  95.1%      --------------------------------------------------------------------------------
  8 YWHAZP7         12.5%  88.4%      --------------------------------------------------------------------------------
  9 YWHAZP8         14.5%  85.2%      --------------------------------------------------------------------------------
 10 YWHAZP10        14.7%  98.0%      --------------------------------------------------------------------------------
    consensus/100%                    ................................................................................
    consensus/90%                     ................................................................................
    consensus/80%                     ................................................................................
    consensus/70%                     ................................................................................

                      cov    pid 2481          .         5         .         .         .         .         :         . 2560
  1 YWHAZ          100.0% 100.0%      TACTGCAGCAAATCTCTCCTTAGTTGATGAGACTGTGTTTATCTCCCTTTAAAACCCTACCTATCCTGAATGGTCTGTCA
  2 YWHAZP1         14.3%  80.6%      --------------------------------------------------------------------------------
  3 YWHAZP2         14.5%  97.1%      --------------------------------------------------------------------------------
  4 YWHAZP3         14.7%  97.6%      --------------------------------------------------------------------------------
  5 YWHAZP4         14.1%  90.5%      --------------------------------------------------------------------------------
  6 YWHAZP5         14.6%  96.0%      --------------------------------------------------------------------------------
```

```
 7 YWHAZP6          14.7%  95.1%       --------------------------------------------------------------------------------
 8 YWHAZP7          12.5%  88.4%       --------------------------------------------------------------------------------
 9 YWHAZP8          14.5%  85.2%       --------------------------------------------------------------------------------
10 YWHAZP10         14.7%  98.0%       --------------------------------------------------------------------------------
   consensus/100%                      ................................................................................
   consensus/90%                       ................................................................................
   consensus/80%                       ................................................................................
   consensus/70%                       ................................................................................

                      cov    pid  2561          .         .         .         6         .         .         .         . 2640
 1 YWHAZ           100.0% 100.0%       TTGTCTGCCTTTAAAATCCTTCCTCTTTCTTCCTCCTCTATTCTCTAAATAATGATGGGGCTAAGTTATACCCAAAGCTC
 2 YWHAZP1          14.3%  80.6%       --------------------------------------------------------------------------------
 3 YWHAZP2          14.5%  97.1%       --------------------------------------------------------------------------------
 4 YWHAZP3          14.7%  97.6%       --------------------------------------------------------------------------------
 5 YWHAZP4          14.1%  90.5%       --------------------------------------------------------------------------------
 6 YWHAZP5          14.6%  96.0%       --------------------------------------------------------------------------------
 7 YWHAZP6          14.7%  95.1%       --------------------------------------------------------------------------------
 8 YWHAZP7          12.5%  88.4%       --------------------------------------------------------------------------------
 9 YWHAZP8          14.5%  85.2%       --------------------------------------------------------------------------------
10 YWHAZP10         14.7%  98.0%       --------------------------------------------------------------------------------
   consensus/100%                      ................................................................................
   consensus/90%                       ................................................................................
   consensus/80%                       ................................................................................
   consensus/70%                       ................................................................................

                      cov    pid  2641          :         .         .         .         .         7         .         . 2720
 1 YWHAZ           100.0% 100.0%       ACTTTACAAAATATTTCCTCAGTACTTTGCAGAAAACACCAAACAAAAATGCCATTTTAAAAAAGGTGTATTTTTTCTTT
 2 YWHAZP1          14.3%  80.6%       --------------------------------------------------------------------------------
 3 YWHAZP2          14.5%  97.1%       --------------------------------------------------------------------------------
 4 YWHAZP3          14.7%  97.6%       --------------------------------------------------------------------------------
 5 YWHAZP4          14.1%  90.5%       --------------------------------------------------------------------------------
 6 YWHAZP5          14.6%  96.0%       --------------------------------------------------------------------------------
 7 YWHAZP6          14.7%  95.1%       --------------------------------------------------------------------------------
 8 YWHAZP7          12.5%  88.4%       --------------------------------------------------------------------------------
 9 YWHAZP8          14.5%  85.2%       --------------------------------------------------------------------------------
10 YWHAZP10         14.7%  98.0%       --------------------------------------------------------------------------------
   consensus/100%                      ................................................................................
   consensus/90%                       ................................................................................
   consensus/80%                       ................................................................................
   consensus/70%                       ................................................................................

                      cov    pid  2721          .         .         :         .         .         .         .         8 2800
 1 YWHAZ           100.0% 100.0%       TAGAATGTAAGCTCCTCAAGAGCAGGGACAATGTTTTCTGTATGTTCTATTGTGCCTAGTACACTGTAAATGCTCAATAA
 2 YWHAZP1          14.3%  80.6%       --------------------------------------------------------------------------------
 3 YWHAZP2          14.5%  97.1%       --------------------------------------------------------------------------------
 4 YWHAZP3          14.7%  97.6%       --------------------------------------------------------------------------------
 5 YWHAZP4          14.1%  90.5%       --------------------------------------------------------------------------------
 6 YWHAZP5          14.6%  96.0%       --------------------------------------------------------------------------------
 7 YWHAZP6          14.7%  95.1%       --------------------------------------------------------------------------------
 8 YWHAZP7          12.5%  88.4%       --------------------------------------------------------------------------------
 9 YWHAZP8          14.5%  85.2%       --------------------------------------------------------------------------------
10 YWHAZP10         14.7%  98.0%       --------------------------------------------------------------------------------
   consensus/100%                      ................................................................................
   consensus/90%                       ................................................................................
   consensus/80%                       ................................................................................
   consensus/70%                       ................................................................................

                      cov    pid  2801          .         .         .         .         :         .         .         . 2880
 1 YWHAZ           100.0% 100.0%       ATATTGATGATGGGAGGCAGTGAGTCTTGATGATAAGGGTGAGAAACTGAAATCCCAAACACTGTTTTGTTGCTTGTTTT
 2 YWHAZP1          14.3%  80.6%       --------------------------------------------------------------------------------
 3 YWHAZP2          14.5%  97.1%       --------------------------------------------------------------------------------
 4 YWHAZP3          14.7%  97.6%       --------------------------------------------------------------------------------
 5 YWHAZP4          14.1%  90.5%       --------------------------------------------------------------------------------
 6 YWHAZP5          14.6%  96.0%       --------------------------------------------------------------------------------
 7 YWHAZP6          14.7%  95.1%       --------------------------------------------------------------------------------
 8 YWHAZP7          12.5%  88.4%       --------------------------------------------------------------------------------
 9 YWHAZP8          14.5%  85.2%       --------------------------------------------------------------------------------
10 YWHAZP10         14.7%  98.0%       --------------------------------------------------------------------------------
   consensus/100%                      ................................................................................
   consensus/90%                       ................................................................................
   consensus/80%                       ................................................................................
   consensus/70%                       ................................................................................

                      cov    pid  2881          .         9         .         .         .         .         :         . 2960
 1 YWHAZ           100.0% 100.0%       ATTATGACCTCAGATTAAATTGGGAAATATTGGCCCTTTTGAATAATTGTCCCAAATATTACATTCAAATAAAAGTGCAA
 2 YWHAZP1          14.3%  80.6%       --------------------------------------------------------------------------------
 3 YWHAZP2          14.5%  97.1%       --------------------------------------------------------------------------------
 4 YWHAZP3          14.7%  97.6%       --------------------------------------------------------------------------------
 5 YWHAZP4          14.1%  90.5%       --------------------------------------------------------------------------------
 6 YWHAZP5          14.6%  96.0%       --------------------------------------------------------------------------------
 7 YWHAZP6          14.7%  95.1%       --------------------------------------------------------------------------------
 8 YWHAZP7          12.5%  88.4%       --------------------------------------------------------------------------------
 9 YWHAZP8          14.5%  85.2%       --------------------------------------------------------------------------------
10 YWHAZP10         14.7%  98.0%       --------------------------------------------------------------------------------
   consensus/100%                      ................................................................................
```

```
    consensus/90%                   ................................................................................
    consensus/80%                   ................................................................................
    consensus/70%                   ................................................................................

                    cov    pid 2961          .         .         .         0         .         .         .         . 3040
  1 YWHAZ        100.0% 100.0%      TGGAGACCTTGGGGTCTTTATTTCCAGGAGAAAAGATACCTTCTGCATCTAGGGCAACAATCCTTTTCCCTCGCAGCTCT
  2 YWHAZP1       14.3%  80.6%      --------------------------------------------------------------------------------
  3 YWHAZP2       14.5%  97.1%      --------------------------------------------------------------------------------
  4 YWHAZP3       14.7%  97.6%      --------------------------------------------------------------------------------
  5 YWHAZP4       14.1%  90.5%      --------------------------------------------------------------------------------
  6 YWHAZP5       14.6%  96.0%      --------------------------------------------------------------------------------
  7 YWHAZP6       14.7%  95.1%      --------------------------------------------------------------------------------
  8 YWHAZP7       12.5%  88.4%      --------------------------------------------------------------------------------
  9 YWHAZP8       14.5%  85.2%      --------------------------------------------------------------------------------
 10 YWHAZP10      14.7%  98.0%      --------------------------------------------------------------------------------
    consensus/100%                  ................................................................................
    consensus/90%                   ................................................................................
    consensus/80%                   ................................................................................
    consensus/70%                   ................................................................................

                    cov    pid 3041          :         .         .         .         .         1         .         . 3120
  1 YWHAZ        100.0% 100.0%      TTCTTTTACCTCACTTACTGATTTCTCCTGACCCCCAAGCCAAGGAGGTAAAATAATTATATATATATATATATATATAT
  2 YWHAZP1       14.3%  80.6%      --------------------------------------------------------------------------------
  3 YWHAZP2       14.5%  97.1%      --------------------------------------------------------------------------------
  4 YWHAZP3       14.7%  97.6%      --------------------------------------------------------------------------------
  5 YWHAZP4       14.1%  90.5%      --------------------------------------------------------------------------------
  6 YWHAZP5       14.6%  96.0%      --------------------------------------------------------------------------------
  7 YWHAZP6       14.7%  95.1%      --------------------------------------------------------------------------------
  8 YWHAZP7       12.5%  88.4%      --------------------------------------------------------------------------------
  9 YWHAZP8       14.5%  85.2%      --------------------------------------------------------------------------------
 10 YWHAZP10      14.7%  98.0%      --------------------------------------------------------------------------------
    consensus/100%                  ................................................................................
    consensus/90%                   ................................................................................
    consensus/80%                   ................................................................................
    consensus/70%                   ................................................................................

                    cov    pid 3121          .         .         :         .         .         .         .         2 3200
  1 YWHAZ        100.0% 100.0%      ATATATATATATAAAGTAATTATATTTTATAGCTAGACTGAAGAGGTGGGAATTTTTTTGTTGTTTTTTTTTTTTTTGGA
  2 YWHAZP1       14.3%  80.6%      --------------------------------------------------------------------------------
  3 YWHAZP2       14.5%  97.1%      --------------------------------------------------------------------------------
  4 YWHAZP3       14.7%  97.6%      --------------------------------------------------------------------------------
  5 YWHAZP4       14.1%  90.5%      --------------------------------------------------------------------------------
  6 YWHAZP5       14.6%  96.0%      --------------------------------------------------------------------------------
  7 YWHAZP6       14.7%  95.1%      --------------------------------------------------------------------------------
  8 YWHAZP7       12.5%  88.4%      --------------------------------------------------------------------------------
  9 YWHAZP8       14.5%  85.2%      --------------------------------------------------------------------------------
 10 YWHAZP10      14.7%  98.0%      --------------------------------------------------------------------------------
    consensus/100%                  ................................................................................
    consensus/90%                   ................................................................................
    consensus/80%                   ................................................................................
    consensus/70%                   ................................................................................

                    cov    pid 3201          .         .         .         .         :         .         .         . 3280
  1 YWHAZ        100.0% 100.0%      GACAAGAGTCTTGCTCTGTCACCCAGGCTTGAGTGCAGTGGTGCGATCTCGGCTGAGTGCAACCTCCGCCTGCTGGGTTC
  2 YWHAZP1       14.3%  80.6%      --------------------------------------------------------------------------------
  3 YWHAZP2       14.5%  97.1%      --------------------------------------------------------------------------------
  4 YWHAZP3       14.7%  97.6%      --------------------------------------------------------------------------------
  5 YWHAZP4       14.1%  90.5%      --------------------------------------------------------------------------------
  6 YWHAZP5       14.6%  96.0%      --------------------------------------------------------------------------------
  7 YWHAZP6       14.7%  95.1%      --------------------------------------------------------------------------------
  8 YWHAZP7       12.5%  88.4%      --------------------------------------------------------------------------------
  9 YWHAZP8       14.5%  85.2%      --------------------------------------------------------------------------------
 10 YWHAZP10      14.7%  98.0%      --------------------------------------------------------------------------------
    consensus/100%                  ................................................................................
    consensus/90%                   ................................................................................
    consensus/80%                   ................................................................................
    consensus/70%                   ................................................................................

                    cov    pid 3281          .         3         .         .         .         .         :         . 3360
  1 YWHAZ        100.0% 100.0%      AAGCGATTCTCCTGCCTCAGCCTCCCAAGTAGCTGGGATTACAGGTGCCCGCCACCACACCTGGCTAATTTTTGTGTTTT
  2 YWHAZP1       14.3%  80.6%      --------------------------------------------------------------------------------
  3 YWHAZP2       14.5%  97.1%      --------------------------------------------------------------------------------
  4 YWHAZP3       14.7%  97.6%      --------------------------------------------------------------------------------
  5 YWHAZP4       14.1%  90.5%      --------------------------------------------------------------------------------
  6 YWHAZP5       14.6%  96.0%      --------------------------------------------------------------------------------
  7 YWHAZP6       14.7%  95.1%      --------------------------------------------------------------------------------
  8 YWHAZP7       12.5%  88.4%      --------------------------------------------------------------------------------
  9 YWHAZP8       14.5%  85.2%      --------------------------------------------------------------------------------
 10 YWHAZP10      14.7%  98.0%      --------------------------------------------------------------------------------
    consensus/100%                  ................................................................................
    consensus/90%                   ................................................................................
    consensus/80%                   ................................................................................
    consensus/70%                   ................................................................................

                    cov    pid 3361          .         .         .         4         .         .         .         . 3440
```

```
 1 YWHAZ          100.0% 100.0%    TAGTAGAGACAGGGTTTCACCATGTTGTCCAGGCTGGTCTTGAATTCCCGACCTCAGGCAATCCGCCCATCTCAACCTCC
 2 YWHAZP1         14.3%  80.6%    --------------------------------------------------------------------------------
 3 YWHAZP2         14.5%  97.1%    --------------------------------------------------------------------------------
 4 YWHAZP3         14.7%  97.6%    --------------------------------------------------------------------------------
 5 YWHAZP4         14.1%  90.5%    --------------------------------------------------------------------------------
 6 YWHAZP5         14.6%  96.0%    --------------------------------------------------------------------------------
 7 YWHAZP6         14.7%  95.1%    --------------------------------------------------------------------------------
 8 YWHAZP7         12.5%  88.4%    --------------------------------------------------------------------------------
 9 YWHAZP8         14.5%  85.2%    --------------------------------------------------------------------------------
10 YWHAZP10        14.7%  98.0%    --------------------------------------------------------------------------------
   consensus/100%                  ................................................................................
   consensus/90%                   ................................................................................
   consensus/80%                   ................................................................................
   consensus/70%                   ................................................................................

                     cov    pid 3441          :         .         .         .         .         5         .         . 3520
 1 YWHAZ          100.0% 100.0%    CAAAGTGCTGGGATTACAGGCATAAGCCACCGCGCCCAGTAGAGATGTGATATTATTCAAGTAAATAAAACCTGAAACTT
 2 YWHAZP1         14.3%  80.6%    --------------------------------------------------------------------------------
 3 YWHAZP2         14.5%  97.1%    --------------------------------------------------------------------------------
 4 YWHAZP3         14.7%  97.6%    --------------------------------------------------------------------------------
 5 YWHAZP4         14.1%  90.5%    --------------------------------------------------------------------------------
 6 YWHAZP5         14.6%  96.0%    --------------------------------------------------------------------------------
 7 YWHAZP6         14.7%  95.1%    --------------------------------------------------------------------------------
 8 YWHAZP7         12.5%  88.4%    --------------------------------------------------------------------------------
 9 YWHAZP8         14.5%  85.2%    --------------------------------------------------------------------------------
10 YWHAZP10        14.7%  98.0%    --------------------------------------------------------------------------------
   consensus/100%                  ................................................................................
   consensus/90%                   ................................................................................
   consensus/80%                   ................................................................................
   consensus/70%                   ................................................................................

                     cov    pid 3521          .         .         :         .         .         .         .         6 3600
 1 YWHAZ          100.0% 100.0%    GGCACATTTGCCTATTTAATGTGCATCAGTCATCTGAACTAGGTTGATCCTGGAATAATGAAAACACTTCACTAGTATTA
 2 YWHAZP1         14.3%  80.6%    --------------------------------------------------------------------------------
 3 YWHAZP2         14.5%  97.1%    --------------------------------------------------------------------------------
 4 YWHAZP3         14.7%  97.6%    --------------------------------------------------------------------------------
 5 YWHAZP4         14.1%  90.5%    --------------------------------------------------------------------------------
 6 YWHAZP5         14.6%  96.0%    --------------------------------------------------------------------------------
 7 YWHAZP6         14.7%  95.1%    --------------------------------------------------------------------------------
 8 YWHAZP7         12.5%  88.4%    --------------------------------------------------------------------------------
 9 YWHAZP8         14.5%  85.2%    --------------------------------------------------------------------------------
10 YWHAZP10        14.7%  98.0%    --------------------------------------------------------------------------------
   consensus/100%                  ................................................................................
   consensus/90%                   ................................................................................
   consensus/80%                   ................................................................................
   consensus/70%                   ................................................................................

                     cov    pid 3601          .         .         .         .         :         .         .         . 3680
 1 YWHAZ          100.0% 100.0%    ACTAATGTTTTCTACATAGTAGTTCCCAATAGATTAAGGAAAATTTCCAACCTGAGATTCTTTGTTTTGGAATTTGAATA
 2 YWHAZP1         14.3%  80.6%    --------------------------------------------------------------------------------
 3 YWHAZP2         14.5%  97.1%    --------------------------------------------------------------------------------
 4 YWHAZP3         14.7%  97.6%    --------------------------------------------------------------------------------
 5 YWHAZP4         14.1%  90.5%    --------------------------------------------------------------------------------
 6 YWHAZP5         14.6%  96.0%    --------------------------------------------------------------------------------
 7 YWHAZP6         14.7%  95.1%    --------------------------------------------------------------------------------
 8 YWHAZP7         12.5%  88.4%    --------------------------------------------------------------------------------
 9 YWHAZP8         14.5%  85.2%    --------------------------------------------------------------------------------
10 YWHAZP10        14.7%  98.0%    --------------------------------------------------------------------------------
   consensus/100%                  ................................................................................
   consensus/90%                   ................................................................................
   consensus/80%                   ................................................................................
   consensus/70%                   ................................................................................

                     cov    pid 3681          .         7         .         .         .         .         :         . 3760
 1 YWHAZ          100.0% 100.0%    ATGTGTTTGTAACTATTCCTTGATTGTCCCAAATCCAGGCTTTACGTCTGCTTTTGTCAATGTAACATCCTAGTGCCAGG
 2 YWHAZP1         14.3%  80.6%    --------------------------------------------------------------------------------
 3 YWHAZP2         14.5%  97.1%    --------------------------------------------------------------------------------
 4 YWHAZP3         14.7%  97.6%    --------------------------------------------------------------------------------
 5 YWHAZP4         14.1%  90.5%    --------------------------------------------------------------------------------
 6 YWHAZP5         14.6%  96.0%    --------------------------------------------------------------------------------
 7 YWHAZP6         14.7%  95.1%    --------------------------------------------------------------------------------
 8 YWHAZP7         12.5%  88.4%    --------------------------------------------------------------------------------
 9 YWHAZP8         14.5%  85.2%    --------------------------------------------------------------------------------
10 YWHAZP10        14.7%  98.0%    --------------------------------------------------------------------------------
   consensus/100%                  ................................................................................
   consensus/90%                   ................................................................................
   consensus/80%                   ................................................................................
   consensus/70%                   ................................................................................

                     cov    pid 3761          .         .         .         8         .         .         .         . 3840
 1 YWHAZ          100.0% 100.0%    AAATTGGAAAATCCAATGACTTTTTGTGTGTAAAGTACTGGAATTTTTTTGTTCGTTTGTTTTTAAGACGGAGTGTCAAC
 2 YWHAZP1         14.3%  80.6%    --------------------------------------------------------------------------------
 3 YWHAZP2         14.5%  97.1%    --------------------------------------------------------------------------------
 4 YWHAZP3         14.7%  97.6%    --------------------------------------------------------------------------------
 5 YWHAZP4         14.1%  90.5%    --------------------------------------------------------------------------------
```

```
 6 YWHAZP5         14.6%  96.0%      --------------------------------------------------------------------------------
 7 YWHAZP6         14.7%  95.1%      --------------------------------------------------------------------------------
 8 YWHAZP7         12.5%  88.4%      --------------------------------------------------------------------------------
 9 YWHAZP8         14.5%  85.2%      --------------------------------------------------------------------------------
10 YWHAZP10        14.7%  98.0%      --------------------------------------------------------------------------------
   consensus/100%                    ................................................................................
   consensus/90%                     ................................................................................
   consensus/80%                     ................................................................................
   consensus/70%                     ................................................................................

                     cov    pid 3841          :         .         .         .         .         9         .         . 3920
 1 YWHAZ          100.0% 100.0%      TCTCGCCCAGGCTGGGGTGCAGTGGCCTGATCTCGGCTCACTGCAACCTTCTCCTCCCAGGCTGAAGTGATTCTCCTGCC
 2 YWHAZP1         14.3%  80.6%      --------------------------------------------------------------------------------
 3 YWHAZP2         14.5%  97.1%      --------------------------------------------------------------------------------
 4 YWHAZP3         14.7%  97.6%      --------------------------------------------------------------------------------
 5 YWHAZP4         14.1%  90.5%      --------------------------------------------------------------------------------
 6 YWHAZP5         14.6%  96.0%      --------------------------------------------------------------------------------
 7 YWHAZP6         14.7%  95.1%      --------------------------------------------------------------------------------
 8 YWHAZP7         12.5%  88.4%      --------------------------------------------------------------------------------
 9 YWHAZP8         14.5%  85.2%      --------------------------------------------------------------------------------
10 YWHAZP10        14.7%  98.0%      --------------------------------------------------------------------------------
   consensus/100%                    ................................................................................
   consensus/90%                     ................................................................................
   consensus/80%                     ................................................................................
   consensus/70%                     ................................................................................

                     cov    pid 3921          .         .         :         .         .         .         .         0 4000
 1 YWHAZ          100.0% 100.0%      TCAGCCTCGCAAGTAGCTGGGACTGCAGGCATGTGCCACCACTCCTGGCTAATTATTTGTATTTTTAGTAGAGAGAGGGT
 2 YWHAZP1         14.3%  80.6%      --------------------------------------------------------------------------------
 3 YWHAZP2         14.5%  97.1%      --------------------------------------------------------------------------------
 4 YWHAZP3         14.7%  97.6%      --------------------------------------------------------------------------------
 5 YWHAZP4         14.1%  90.5%      --------------------------------------------------------------------------------
 6 YWHAZP5         14.6%  96.0%      --------------------------------------------------------------------------------
 7 YWHAZP6         14.7%  95.1%      --------------------------------------------------------------------------------
 8 YWHAZP7         12.5%  88.4%      --------------------------------------------------------------------------------
 9 YWHAZP8         14.5%  85.2%      --------------------------------------------------------------------------------
10 YWHAZP10        14.7%  98.0%      --------------------------------------------------------------------------------
   consensus/100%                    ................................................................................
   consensus/90%                     ................................................................................
   consensus/80%                     ................................................................................
   consensus/70%                     ................................................................................

                     cov    pid 4001          .         .         .         .         :         .         .         . 4080
 1 YWHAZ          100.0% 100.0%      TTCACCGTGTTAGCCATGATGGTCTCATTCTCCTGACCTTGTGATCCGCCCACCTCGGCCTCCCAAAGTGCTGGGATTAC
 2 YWHAZP1         14.3%  80.6%      --------------------------------------------------------------------------------
 3 YWHAZP2         14.5%  97.1%      --------------------------------------------------------------------------------
 4 YWHAZP3         14.7%  97.6%      --------------------------------------------------------------------------------
 5 YWHAZP4         14.1%  90.5%      --------------------------------------------------------------------------------
 6 YWHAZP5         14.6%  96.0%      --------------------------------------------------------------------------------
 7 YWHAZP6         14.7%  95.1%      --------------------------------------------------------------------------------
 8 YWHAZP7         12.5%  88.4%      --------------------------------------------------------------------------------
 9 YWHAZP8         14.5%  85.2%      --------------------------------------------------------------------------------
10 YWHAZP10        14.7%  98.0%      --------------------------------------------------------------------------------
   consensus/100%                    ................................................................................
   consensus/90%                     ................................................................................
   consensus/80%                     ................................................................................
   consensus/70%                     ................................................................................

                     cov    pid 4081          .         1         .         .         .         .         :         . 4160
 1 YWHAZ          100.0% 100.0%      AGGCGTGAGCCACTGTGCCCACCCTGGTATGTTTTTTTTAAATTGGCCACTTAAGAGTTGTCCTTCAGAAAGTGGAGCAG
 2 YWHAZP1         14.3%  80.6%      --------------------------------------------------------------------------------
 3 YWHAZP2         14.5%  97.1%      --------------------------------------------------------------------------------
 4 YWHAZP3         14.7%  97.6%      --------------------------------------------------------------------------------
 5 YWHAZP4         14.1%  90.5%      --------------------------------------------------------------------------------
 6 YWHAZP5         14.6%  96.0%      --------------------------------------------------------------------------------
 7 YWHAZP6         14.7%  95.1%      --------------------------------------------------------------------------------
 8 YWHAZP7         12.5%  88.4%      --------------------------------------------------------------------------------
 9 YWHAZP8         14.5%  85.2%      --------------------------------------------------------------------------------
10 YWHAZP10        14.7%  98.0%      --------------------------------------------------------------------------------
   consensus/100%                    ................................................................................
   consensus/90%                     ................................................................................
   consensus/80%                     ................................................................................
   consensus/70%                     ................................................................................

                     cov    pid 4161          .         .         .         2         .         .         .         . 4240
 1 YWHAZ          100.0% 100.0%      CAGTGTGATTAGTAAAAACGATGGGCTTTCCTGCAGCTCTGGCTTATGGTCTTTGTTTTTCCAGTTAACAGCTGTTACTT
 2 YWHAZP1         14.3%  80.6%      --------------------------------------------------------------------------------
 3 YWHAZP2         14.5%  97.1%      --------------------------------------------------------------------------------
 4 YWHAZP3         14.7%  97.6%      --------------------------------------------------------------------------------
 5 YWHAZP4         14.1%  90.5%      --------------------------------------------------------------------------------
 6 YWHAZP5         14.6%  96.0%      --------------------------------------------------------------------------------
 7 YWHAZP6         14.7%  95.1%      --------------------------------------------------------------------------------
 8 YWHAZP7         12.5%  88.4%      --------------------------------------------------------------------------------
 9 YWHAZP8         14.5%  85.2%      --------------------------------------------------------------------------------
10 YWHAZP10        14.7%  98.0%      --------------------------------------------------------------------------------
```

```
    consensus/100%                    ................................................................................
    consensus/90%                     ................................................................................
    consensus/80%                     ................................................................................
    consensus/70%                     ................................................................................

                      cov    pid 4241          :         .         .         .         .         3         .         . 4320
  1 YWHAZ          100.0% 100.0%      CATGCTGTTATAGGGGGTGGGGGGAGAAAGAGAAAGAATATGTCTAGTGGCTTAATTTTGGGGCTCAATTTGTTTATTAT
  2 YWHAZP1         14.3%  80.6%      --------------------------------------------------------------------------------
  3 YWHAZP2         14.5%  97.1%      --------------------------------------------------------------------------------
  4 YWHAZP3         14.7%  97.6%      --------------------------------------------------------------------------------
  5 YWHAZP4         14.1%  90.5%      --------------------------------------------------------------------------------
  6 YWHAZP5         14.6%  96.0%      --------------------------------------------------------------------------------
  7 YWHAZP6         14.7%  95.1%      --------------------------------------------------------------------------------
  8 YWHAZP7         12.5%  88.4%      --------------------------------------------------------------------------------
  9 YWHAZP8         14.5%  85.2%      --------------------------------------------------------------------------------
 10 YWHAZP10        14.7%  98.0%      --------------------------------------------------------------------------------
    consensus/100%                    ................................................................................
    consensus/90%                     ................................................................................
    consensus/80%                     ................................................................................
    consensus/70%                     ................................................................................

                      cov    pid 4321          .         .         :         .         .         .         .         4 4400
  1 YWHAZ          100.0% 100.0%      GTTTTTCCTGCAAAAGAGTCACCAGTCTTGTCATGGCTCAATGCATATTTTAAAATGAGTCAAAGCCAATTTGGAAGCTG
  2 YWHAZP1         14.3%  80.6%      --------------------------------------------------------------------------------
  3 YWHAZP2         14.5%  97.1%      --------------------------------------------------------------------------------
  4 YWHAZP3         14.7%  97.6%      --------------------------------------------------------------------------------
  5 YWHAZP4         14.1%  90.5%      --------------------------------------------------------------------------------
  6 YWHAZP5         14.6%  96.0%      --------------------------------------------------------------------------------
  7 YWHAZP6         14.7%  95.1%      --------------------------------------------------------------------------------
  8 YWHAZP7         12.5%  88.4%      --------------------------------------------------------------------------------
  9 YWHAZP8         14.5%  85.2%      --------------------------------------------------------------------------------
 10 YWHAZP10        14.7%  98.0%      --------------------------------------------------------------------------------
    consensus/100%                    ................................................................................
    consensus/90%                     ................................................................................
    consensus/80%                     ................................................................................
    consensus/70%                     ................................................................................

                      cov    pid 4401          .         .         .         .         :         .         .         . 4480
  1 YWHAZ          100.0% 100.0%      CTATCTTAGGTTATTTGTAGTCAGGCTTCAAATGGAGAGTGAAACTTTGACTTTGAAAACTGGTGCTCTATGCTCAATGA
  2 YWHAZP1         14.3%  80.6%      --------------------------------------------------------------------------------
  3 YWHAZP2         14.5%  97.1%      --------------------------------------------------------------------------------
  4 YWHAZP3         14.7%  97.6%      --------------------------------------------------------------------------------
  5 YWHAZP4         14.1%  90.5%      --------------------------------------------------------------------------------
  6 YWHAZP5         14.6%  96.0%      --------------------------------------------------------------------------------
  7 YWHAZP6         14.7%  95.1%      --------------------------------------------------------------------------------
  8 YWHAZP7         12.5%  88.4%      --------------------------------------------------------------------------------
  9 YWHAZP8         14.5%  85.2%      --------------------------------------------------------------------------------
 10 YWHAZP10        14.7%  98.0%      --------------------------------------------------------------------------------
    consensus/100%                    ................................................................................
    consensus/90%                     ................................................................................
    consensus/80%                     ................................................................................
    consensus/70%                     ................................................................................

                      cov    pid 4481          .         5         .         .         .         .         :         . 4560
  1 YWHAZ          100.0% 100.0%      TGGTCTTACACATTCCTCTAGGGAAAGGTCAAGAAATAAATTTGGCTTGATTGTATTCTCTCATTTACCATATAGGAAAT
  2 YWHAZP1         14.3%  80.6%      --------------------------------------------------------------------------------
  3 YWHAZP2         14.5%  97.1%      --------------------------------------------------------------------------------
  4 YWHAZP3         14.7%  97.6%      --------------------------------------------------------------------------------
  5 YWHAZP4         14.1%  90.5%      --------------------------------------------------------------------------------
  6 YWHAZP5         14.6%  96.0%      --------------------------------------------------------------------------------
  7 YWHAZP6         14.7%  95.1%      --------------------------------------------------------------------------------
  8 YWHAZP7         12.5%  88.4%      --------------------------------------------------------------------------------
  9 YWHAZP8         14.5%  85.2%      --------------------------------------------------------------------------------
 10 YWHAZP10        14.7%  98.0%      --------------------------------------------------------------------------------
    consensus/100%                    ................................................................................
    consensus/90%                     ................................................................................
    consensus/80%                     ................................................................................
    consensus/70%                     ................................................................................

                      cov    pid 4561          .         .         .         6         .         .         .         . 4640
  1 YWHAZ          100.0% 100.0%      ACTATGGTAGAACTGAAAATATGTACAATAGTAAAGTGGTGGCTGAGACTGGGCACTTGGAAAATAGACTTGGATTCTTT
  2 YWHAZP1         14.3%  80.6%      --------------------------------------------------------------------------------
  3 YWHAZP2         14.5%  97.1%      --------------------------------------------------------------------------------
  4 YWHAZP3         14.7%  97.6%      --------------------------------------------------------------------------------
  5 YWHAZP4         14.1%  90.5%      --------------------------------------------------------------------------------
  6 YWHAZP5         14.6%  96.0%      --------------------------------------------------------------------------------
  7 YWHAZP6         14.7%  95.1%      --------------------------------------------------------------------------------
  8 YWHAZP7         12.5%  88.4%      --------------------------------------------------------------------------------
  9 YWHAZP8         14.5%  85.2%      --------------------------------------------------------------------------------
 10 YWHAZP10        14.7%  98.0%      --------------------------------------------------------------------------------
    consensus/100%                    ................................................................................
    consensus/90%                     ................................................................................
    consensus/80%                     ................................................................................
    consensus/70%                     ................................................................................
```

```
                            cov    pid 4641          :         .         .         .         .         7         .         . 4720
 1 YWHAZ                 100.0% 100.0%      TCTAAGTGTGAAACTAATTAAATACTTTTATGTGACTGGTAGTAGTCACAGTCTCCTTGATTGATTCTAGTTCCTTTTCA
 2 YWHAZP1                14.3%  80.6%      --------------------------------------------------------------------------------
 3 YWHAZP2                14.5%  97.1%      --------------------------------------------------------------------------------
 4 YWHAZP3                14.7%  97.6%      --------------------------------------------------------------------------------
 5 YWHAZP4                14.1%  90.5%      --------------------------------------------------------------------------------
 6 YWHAZP5                14.6%  96.0%      --------------------------------------------------------------------------------
 7 YWHAZP6                14.7%  95.1%      --------------------------------------------------------------------------------
 8 YWHAZP7                12.5%  88.4%      --------------------------------------------------------------------------------
 9 YWHAZP8                14.5%  85.2%      --------------------------------------------------------------------------------
10 YWHAZP10               14.7%  98.0%      --------------------------------------------------------------------------------
   consensus/100%                           ................................................................................
   consensus/90%                            ................................................................................
   consensus/80%                            ................................................................................
   consensus/70%                            ................................................................................

                            cov    pid 4721          .         .         :         .         .         .         .         8 4800
 1 YWHAZ                 100.0% 100.0%      AAGTGGAGAGACCTGAATATAGATTGTAACAGGATGTCCAACCTTGCCAGACAAGCAGGCATATATGTCTCTGGGATGCA
 2 YWHAZP1                14.3%  80.6%      --------------------------------------------------------------------------------
 3 YWHAZP2                14.5%  97.1%      --------------------------------------------------------------------------------
 4 YWHAZP3                14.7%  97.6%      --------------------------------------------------------------------------------
 5 YWHAZP4                14.1%  90.5%      --------------------------------------------------------------------------------
 6 YWHAZP5                14.6%  96.0%      --------------------------------------------------------------------------------
 7 YWHAZP6                14.7%  95.1%      --------------------------------------------------------------------------------
 8 YWHAZP7                12.5%  88.4%      --------------------------------------------------------------------------------
 9 YWHAZP8                14.5%  85.2%      --------------------------------------------------------------------------------
10 YWHAZP10               14.7%  98.0%      --------------------------------------------------------------------------------
   consensus/100%                           ................................................................................
   consensus/90%                            ................................................................................
   consensus/80%                            ................................................................................
   consensus/70%                            ................................................................................

                            cov    pid 4801          .         .         .         .         :         .         .         . 4880
 1 YWHAZ                 100.0% 100.0%      GAAGCAGTTACAAGTTAATATATTTAAGCATAATGTTAGCCAATCCTAGGTGATACCAGAGATGTGAATATTTGATCATA
 2 YWHAZP1                14.3%  80.6%      --------------------------------------------------------------------------------
 3 YWHAZP2                14.5%  97.1%      --------------------------------------------------------------------------------
 4 YWHAZP3                14.7%  97.6%      --------------------------------------------------------------------------------
 5 YWHAZP4                14.1%  90.5%      --------------------------------------------------------------------------------
 6 YWHAZP5                14.6%  96.0%      --------------------------------------------------------------------------------
 7 YWHAZP6                14.7%  95.1%      --------------------------------------------------------------------------------
 8 YWHAZP7                12.5%  88.4%      --------------------------------------------------------------------------------
 9 YWHAZP8                14.5%  85.2%      --------------------------------------------------------------------------------
10 YWHAZP10               14.7%  98.0%      --------------------------------------------------------------------------------
   consensus/100%                           ................................................................................
   consensus/90%                            ................................................................................
   consensus/80%                            ................................................................................
   consensus/70%                            ................................................................................

                            cov    pid 4881          .         9         .         .         .         .         :         . 4960
 1 YWHAZ                 100.0% 100.0%      TTTGAGTTCATTCCTTGAAAGTCCAAACCTAACTGGTCTTAATTGATTACTTCTTAGTATTCCGAATTTTAGAATTTAAA
 2 YWHAZP1                14.3%  80.6%      --------------------------------------------------------------------------------
 3 YWHAZP2                14.5%  97.1%      --------------------------------------------------------------------------------
 4 YWHAZP3                14.7%  97.6%      --------------------------------------------------------------------------------
 5 YWHAZP4                14.1%  90.5%      --------------------------------------------------------------------------------
 6 YWHAZP5                14.6%  96.0%      --------------------------------------------------------------------------------
 7 YWHAZP6                14.7%  95.1%      --------------------------------------------------------------------------------
 8 YWHAZP7                12.5%  88.4%      --------------------------------------------------------------------------------
 9 YWHAZP8                14.5%  85.2%      --------------------------------------------------------------------------------
10 YWHAZP10               14.7%  98.0%      --------------------------------------------------------------------------------
   consensus/100%                           ................................................................................
   consensus/90%                            ................................................................................
   consensus/80%                            ................................................................................
   consensus/70%                            ................................................................................

                            cov    pid 4961          .         .         .         0         .        ] 5019
 1 YWHAZ                 100.0% 100.0%      ACCCTATGAATTTTCAGTTTGTGCTTACATTTTCTAACATTGGATGTTTGCTTTGGCCA
 2 YWHAZP1                14.3%  80.6%      -----------------------------------------------------------
 3 YWHAZP2                14.5%  97.1%      -----------------------------------------------------------
 4 YWHAZP3                14.7%  97.6%      -----------------------------------------------------------
 5 YWHAZP4                14.1%  90.5%      -----------------------------------------------------------
 6 YWHAZP5                14.6%  96.0%      -----------------------------------------------------------
 7 YWHAZP6                14.7%  95.1%      -----------------------------------------------------------
 8 YWHAZP7                12.5%  88.4%      -----------------------------------------------------------
 9 YWHAZP8                14.5%  85.2%      -----------------------------------------------------------
10 YWHAZP10               14.7%  98.0%      -----------------------------------------------------------
   consensus/100%                           ...........................................................
   consensus/90%                            ...........................................................
   consensus/80%                            ...........................................................
   consensus/70%                            ...........................................................
```

MView 1.63, Copyright © 1997-2018 Nigel P. Brown

Gene	PAS Score	Location	Poly A Tail Likely
YWHAZP1	1.589	1716	Yes
YWHAZP2	1.951	1747	Yes
YWHAZP3	2.049	1753	Yes
YWHAZP4	2.936	2054	Yes
YWHAZP5	1.634	421	No
YWHAZP6	-0.288	463	No
YWHAZP7	2.154	1489	Yes
YWHAZP8	1.088	1506	Yes
YWHAZP9	2.754	3548	Yes
YWHAZP10	1.213	1750	Yes

Fig S3: PAS score and polyadenylation presence in the pseudogene transcripts.

Gene	Expected Amino Acid Sequence
YWHAZ	MDKNELVQKAKLAEQAERYDDMAACMKSVTEQGAEL SNEERNLLSVAYKNVVGARRSSWRVVSSIEQKTEGAEKKQQMAREYREKIETELRDICNDVLSLLEKFLIPNASQAE<br>SKVFYLKMKGDYYRYLAEVAAGDDKKGIVDQSQQAYQ EAFEISKKEMQPTHPIRLGLALNFSVFYYEILNSPEKACSL<br>AKTAFDEAIAELDTLSEESYKDSTLIMQLLRDNLTLWTSDTQGDEAEAGEGGEN
YWHAZP2	DKNELVQKAKLAEQAEQYDDMAACMKSVTKQGAELSNEERNLLSVAYKNVVGARKSSWRVVSSIEQKTEGAEKKQQMAREHREKIETELRDICNDVLSLLEKFLIPNASQAESKVFYLKMKG<br>DYYRYLTEVTAGDDKIGIVDQSQQAYQEAFEISKKEMQPTHPVRLGLALNFCVLL
YWHAZP3	MDKNELVQKAKLAEQAERYDDMAACMKSVTEQGAELSNEERNLLSVAYKNVVGAHRSSWRVVSSIEQKTEGAEKKQQMAREYREKIETELRDICNDVLSLLEKFLIPSASQAESKVFYLKMEG<br>DYYRYLAEVAAGDDKKGIVDQSQQAYQEAFEISKKEMQPTHPIRLGLALNFSVFYYEILNSPEKACSLAKTAFDEAIAELDTLSEESYKDSTLIMQLLRDNLTLWTSDTQADEGEAGEGVEN
YWHAZP4	LSKELKLSNEERKLLSVASKNVGAYRSFWSVVSSIEQKTEGAEKKQQMAREYREKIQTELRDICNDVLSLLEKFLIPSASQAESKVFYLKMKGDYYRYLAEVVAGDDKKGIVDQSQQAYQEAFEIS<br>KKEMQPTHAIRLDLLL
YWHAZP5	MEKNELVQKAKLAEQAEQYDNMAACMKSVTEQGAELSNEERNLLSVAYKNVVGARRSSWRVISSIEQKTEGVEKKQQMAREYREKIEMELRDISNDVLSLLEKFLISNASQAESKVFYLKMK<br>GDYYRYLAEVAAGDGKKGIVGQSQQAYQEAFEISKKEMQPTHTVRLGLALNFSCSIMRF
YWHAZP6	MDKNELVQKAKLAEQAEWYDDMAACMKSVTEQGA
YWHAZP7	LEKFLIPSASQAESKVFYLKMKGDYYGYLSEVAAGDHKKGIVDQSQQAYQEAFGISKKEMQRAHPIRLGLALNYSVFYYEILNSPRESLLSCKDSCQ
YWHAZP8	LVQKAKLMEQAEKYDDMAACMKSITEQGDELSKEERNLLSITYKIVEGAH
YWHAZP10	MDKNELVQKAKLAEQAERYDDMAACMKSVTEQGAELSNEERNLLSVAYKNVLGARRSSWRVVSSIEQKTEGAEKKQQMAREYREKVDTELRDICNDVLFLLEKFLIPSASQAESKVFSLKMK<br>GDYYHYLAEVATGDDKKGIVDQSQQAYQEAFEISKKEMQPTHPIRPGLALNFSVFYYEILNSPEKACSLAKTAFDEAIAELDTLIEESYKDSMLIMQLLRDNLTLWTSDTQGDEAEAGEGGEN

Fig S4: Predicted amino acid sequence of pseudogene-derived proteins

Fig S5: Amino Acid Alignment [obtained using MView, Reference #29]

```
Reference sequence (1): YWHAZ
Identities normalised by aligned length.
Colored by: identity
```

Key:

u=tiny, s=small, p=polar residues
h=hydrophobic, l=aliphathic, c=charged,
+=positive, - = negative, t=turnlike, u=tiny
a=aromatic

cov = percent coverage of YWHAZ sequence
pid = percent identity in covered regions

```
                        cov     pid   1 [        .         .         .         .         :         .         .         . 80
1 YWHAZ              100.0% 100.0%      MDKNELVQKAKLAEQAERYDDMAACMKSVTEQGAELSNEERNLLSVAYKNVVGARRSSWRVVSSIEQKTEGAEKKQQMAR
2 YWHAZP2             72.2%  93.8%      -DKNELVQKAKLAEQAEQYDDMAACMKSVTKQGAELSNEERNLLSVAYKNVVGARKSSWRVVSSIEQKTEGAEKKQQMAR
3 YWHAZP3            100.0%  97.6%      MDKNELVQKAKLAEQAERYDDMAACMKSVTEQGAELSNEERNLLSVAYKNVVGAHRSSWRVVSSIEQKTEGAEKKQQMAR
4 YWHAZP4             58.0%  86.7%      -----------------------------LSKELKLSNEERKLLSVASKNVG-AYRSFWSVVSSIEQKTEGAEKKQQMAR
5 YWHAZP5             73.9%  90.1%      MEKNELVQKAKLAEQAEQYDNMAACMKSVTEQGAELSNEERNLLSVAYKNVVGARRSSWRVISSIEQKTEGVEKKQQMAR
6 YWHAZP6             13.9%  97.1%      MDKNELVQKAKLAEQAEWYDDMAACMKSVTEQGA----------------------------------------------
7 YWHAZP7             39.6%  80.4%      --------------------------------------------------------------------------------
8 YWHAZP8             23.3%  80.7%      -----LVQKAKLMEQAEKYDDMAACMKSITEQGDELSKEERNLLSITYKIVEGAHRSSWRVF------------------
9 YWHAZP10           100.0%  95.5%      MDKNELVQKAKLAEQAERYDDMAACMKSVTEQGAELSNEERNLLSVAYKNVLGARRSSWRVVSSIEQKTEGAEKKQQMAR
  consensus/100%                        ................................................................................
  consensus/90%                         ................................................................................
  consensus/80%                         .............................hppt...............................................
  consensus/70%                         .....LVQKAKLhEQAE.YDsMAACMKSlTcQGscLSpEERpLLSls.K.V..Ah+S.WpVh..................

                        cov     pid  81          .         1         .         .         .         .         :         . 160
1 YWHAZ              100.0% 100.0%      EYREKIETELRDICNDVLSLLEKFLIPNASQAESKVFYLKMKGDYYRYLAEVAAGDDKKGIVDQSQQAYQEAFEISKKEM
2 YWHAZP2             72.2%  93.8%      EHREKIETELRDICNDVLSLLEKFLIPNASQAESKVFYLKMKGDYYRYLTEVTAGDDKIGIVDQSQQAYQEAFEISKKEM
3 YWHAZP3            100.0%  97.6%      EYREKIETELRDICNDVLSLLEKFLIPSASQAESKVFYLKMEGDYYRYLAEVAAGDDKKGIVDQSQQAYQEAFEISKKEM
4 YWHAZP4             58.0%  86.7%      EYREKIQTELRDICNDVLSLLEKFLIPSASQAESKVFYLKMKGDYYRYLAEVVAGDDKKGIVDQSQQAYQEAFEISKKEM
5 YWHAZP5             73.9%  90.1%      EYREKIEMELRDISNDVLSLLEKFLISNASQAESKVFYLKMKGDYYRYLAEVAAGDGKKGIVGQSQQAYQEAFEISKKEM
6 YWHAZP6             13.9%  97.1%      --------------------------------------------------------------------------------
7 YWHAZP7             39.6%  80.4%      --------------------LEKFLIPSASQAESKVFYLKMKGDYYGYLSEVAAGDHKKGIVDQSQQAYQEAFGISKKEM
8 YWHAZP8             23.3%  80.7%      --------------------------------------------------------------------------------
9 YWHAZP10           100.0%  95.5%      EYREKVDTELRDICNDVLFLLEKFLIPSASQAESKVFSLKMKGDYYHYLAEVATGDDKKGIVDQSQQAYQEAFEISKKEM
  consensus/100%                        ................................................................................
  consensus/90%                         ................................................................................
  consensus/80%                         ................................................................................
  consensus/70%                         ....................LEKFLIssASQAESKVF.LKMcGDYYtYLsEVssGDtKhGIVsQSQQAYQEAFtISKKEM

                        cov     pid 161          .         .         .         2         .         .         .         . 240
1 YWHAZ              100.0% 100.0%      QPTHPIRLGLALNFSVFYYEILNSPEKACSLAKTAFDEAIAELDTLSEESYKDSTLIMQLLRDNLTLWTSDTQGDEAEAG
2 YWHAZP2             72.2%  93.8%      QPTHPVRLGLALNFCVLL--------------------------------------------------------------
3 YWHAZP3            100.0%  97.6%      QPTHPIRLGLALNFSVFYYEILNSPEKACSLAKTAFDEAIAELDTLSEESYKDSTLIMQLLRDNLTLWTSDTQADEGEAG
4 YWHAZP4             58.0%  86.7%      QPTHAIRLDLLL--------------------------------------------------------------------
5 YWHAZP5             73.9%  90.1%      QPTHTVRLGLALNFSCSIMRF-----------------------------------------------------------
6 YWHAZP6             13.9%  97.1%      --------------------------------------------------------------------------------
7 YWHAZP7             39.6%  80.4%      QRAHPIRLGLALNYSVFYYEILNSPRESLLSCKDSCQ-------------------------------------------
8 YWHAZP8             23.3%  80.7%      --------------------------------------------------------------------------------
9 YWHAZP10           100.0%  95.5%      QPTHPIRPGLALNFSVFYYEILNSPEKACSLAKTAFDEAIAELDTLIEESYKDSMLIMQLLRDNLTLWTSDTQGDEAEAG
  consensus/100%                        ................................................................................
  consensus/90%                         ................................................................................
  consensus/80%                         ................................................................................
  consensus/70%                         Q.sHslR.sLhL....................................................................

                        cov     pid 241     ] 245
1 YWHAZ              100.0% 100.0%      EGGEN
2 YWHAZP2             72.2%  93.8%      -----
3 YWHAZP3            100.0%  97.6%      EGVEN
4 YWHAZP4             58.0%  86.7%      -----
5 YWHAZP5             73.9%  90.1%      -----
6 YWHAZP6             13.9%  97.1%      -----
7 YWHAZP7             39.6%  80.4%      -----
8 YWHAZP8             23.3%  80.7%      -----
9 YWHAZP10           100.0%  95.5%      EGGEN
  consensus/100%                        .....
  consensus/90%                         .....
  consensus/80%                         .....
  consensus/70%                         .....
```

MView 1.63, Copyright © 1997-2018 Nigel P. Brown

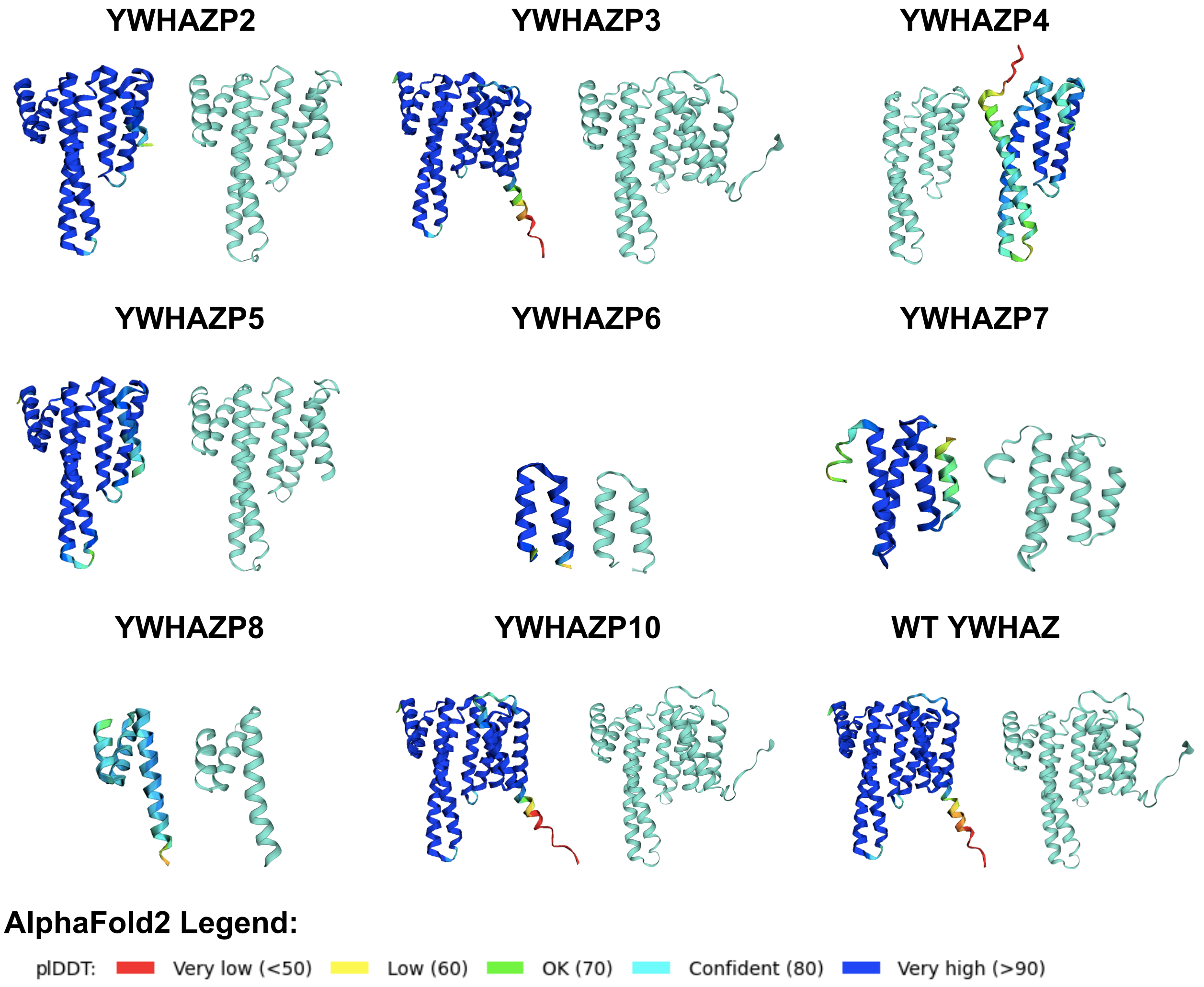

Fig S6:
This figure displays a comparison of the structures generated by AlphaFold2 on the left and SWISS-PROT on the right. AlphaFold2 by DeepMind was used to model potential YWHAZ pseudogene proteins derived from the cDNA sequence on the left. These structures have are colored dark blue and the confidence of each residue is labeled on the structure based upon the plDDT confidence values with a legend above. The SWISS-PROT structure models are colored in turquoise and are on the right of each pair. The SWISS-PROT models were also created based upon the cDNA sequences available for each pseudogene.

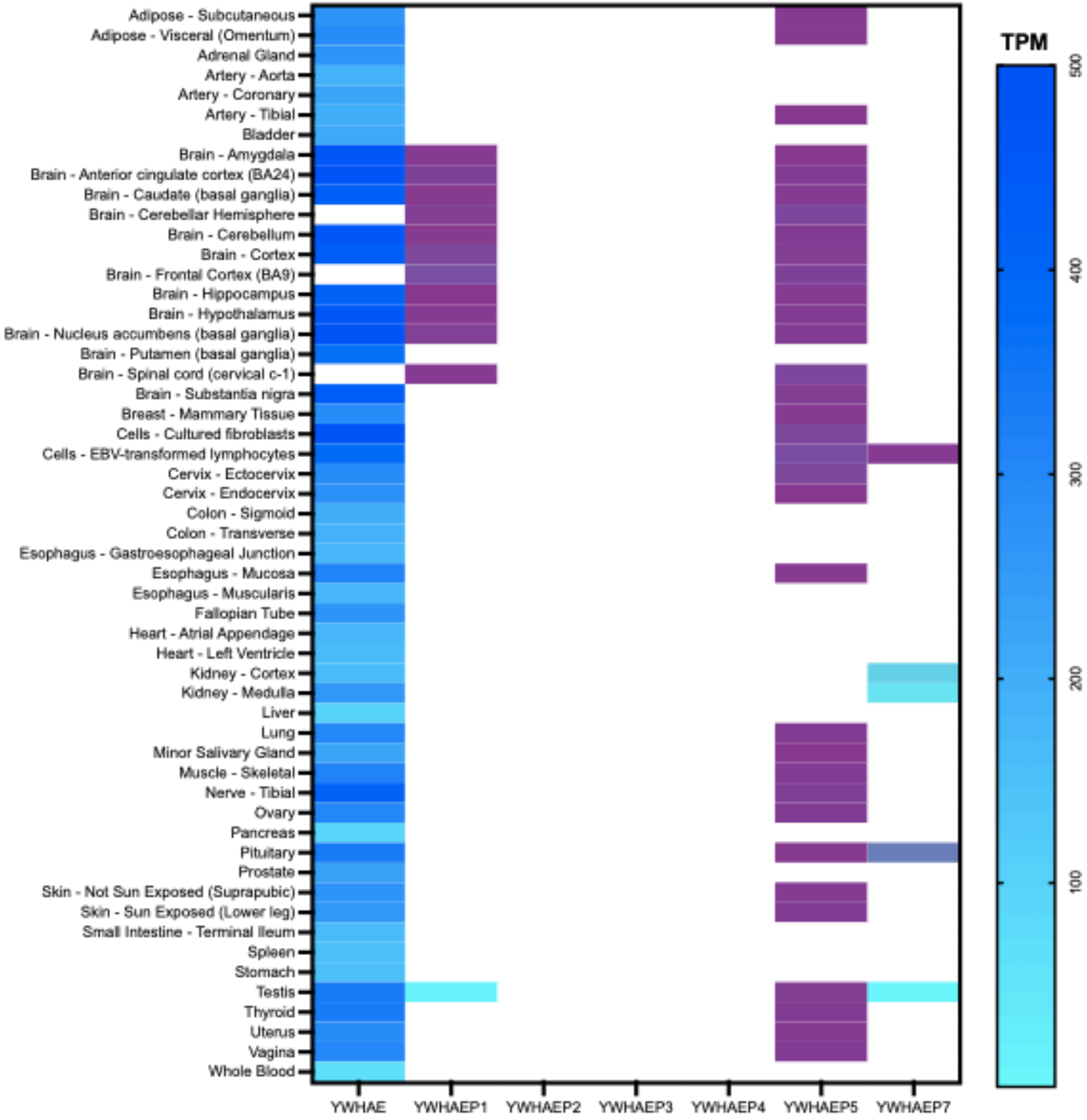

Fig S7. Expression of YWHAE pseudogenes relative to the original gene in different tissues.
The GTEx dataset is used to obtain a heatmap showing baseline pseudogene expression in various tissues and cells in transcripts per million (TPM). The white color indicates no transcripts detected; purple indicates low but detectable transcripts in a specific tissue between 0.1 and 1 TPM; blue indicates that a transcript is detected with the specific transcription level indicated by the darkness of the blue.
